# A 4/8 Subtype α-Conotoxin Vt1.27 Inhibits N-Type Calcium Channels With Potent Anti-Allodynic Effect

**DOI:** 10.3389/fphar.2022.881732

**Published:** 2022-04-29

**Authors:** Shuo Wang, Peter Bartels, Cong Zhao, Arsalan Yousuf, Zhuguo Liu, Shuo Yu, Anuja R. Bony, Xiaoli Ma, Qin Dai, Ting Sun, Na Liu, Mengke Yang, Rilei Yu, Weihong Du, David J. Adams, Qiuyun Dai

**Affiliations:** ^1^ Beijing Institute of Biotechnology, Beijing, China; ^2^ Illawarra Health and Medical Research Institute (IHMRI), University of Wollongong, Wollongong, NSW, Australia; ^3^ Department of Chemistry, Renmin University of China, Beijing, China; ^4^ Department of Pharmacy, PLA Rocket Force Characteristic Medical Center, Beijing, China; ^5^ School of Medicine and Pharmacy, Ocean University of China, Qingdao, China

**Keywords:** *Conus vitulinus*, α-conotoxin Vt1.27, N-type calcium channels, nicotinic acethylcholine receptor, electrophysiology, anti-allodynic effect

## Abstract

A novel 4/8 subtype α-conotoxin, Vt1.27 (NCCMFHTCPIDYSRFNC-NH_2_), was identified from *Conus vitulinus* in the South China Sea by RACE methods. The peptide was synthesized and structurally characterized. Similar to other α-conotoxins that target neuronal nicotinic acetylcholine receptor (nAChR) subtypes, Vt1.27 inhibited the rat α3β2 nAChR subtype (IC_50_ = 1160 nM) and was inactive at voltage-gated sodium and potassium channels in rat sensory neurons. However, Vt1.27 inhibited high voltage-activated N-type (Ca_V_2.2) calcium channels expressed in HEK293T cells with an IC_50_ of 398 nM. An alanine scan of the peptide showed that residues Phe^5^, Pro^9^, Ile^10^, and Ser^13^ contribute significantly to the inhibitory activity of Vt1.27. The molecular dockings indicate that Vt1.27 inhibits the transmembrane region of Ca_V_2.2, which is different from that of ω-conotoxins. Furthermore, Vt1.27 exhibited potent anti-allodynic effect in rat partial sciatic nerve injury (PNL) and chronic constriction injury (CCI) pain models at 10 nmol/kg level with the intramuscular injection. The pain threshold elevation of Vt1.27 groups was higher than that of α-conotoxin Vc1.1 in CCI rat models. These findings expand our knowledge of targets of α-conotoxins and potentially provide a potent, anti-allodynic peptide for the treatment of neuropathic pain.

## Introduction

Chronic pain includes inflammatory and neuropathic pain and becomes a major clinical, social and economic problem. The prevalence of chronic pain ranges from 8% to more than 50% of the population worldwide (Phillips, 2006; Gaskin and Richard, 2012). The development and maintenance of chronic pain are involved in several pathophysiological and maladaptive mechanisms (Alles and Smith, 2018; St John Smith et al., 2018; Finnerup et al., 2021). Several membrane receptors and ion channels have been implicated in pain modulation and chronic pain states. Among these receptors and ion channels, N-type voltage-gated calcium (Ca_V_2.2) channels and neuronal acetylcholine receptors (nAChRs) are related to chronic pain modulation (Pexton et al., 2011; Adams and Berecki, 2013; Patel et al., 2018). Ca_V_2.2 channels consist of a pore-forming α1b subunit and auxiliary β and α2δ subunits which gather in the presynaptic nerve terminals in lamina I and II of the dorsal horn of the spinal cord and are prominently overexpressed in response to injury that induces chronic neuropathic pain (Adams and Berecki, 2013). The nAChRs are pentameric, ligand-gated ion channels and several α subunits (α2-α10) in combination with β subunits (β2-β4) generate a great diversity of nAChRs exhibiting different pharmacological properties (Pexton et al., 2011; Patel et al., 2018). Neuronal nAChRs are involved in various functions including pain sensation, learning, memory, development, and a potential therapeutic target for the treatment of chronic pain (Naser and Kuner, 2018; Nicke et al., 2020).

Marine-derived products are important sources of drugs and nutrient substances, and many of them have entered clinical trials (Di Cesare Mannelli et al., 2021). Conopeptides (conotoxins), secreted from the venom gland of marine cone snails, are largely small peptides that target different types of membrane receptors and ion channels. To date, more than 26 conotoxins superfamilies (A, B, C, D, E, I, M, O, P, S, T, et al.) have been identified (Lavergne et al., 2013; Mir et al., 2016; Jin et al., 2019). α-Conotoxins belong to the A-superfamily and generally consist of 9–20 amino acids with two disulfide bridges. According to the number of amino acids between cysteines (-CCX_n_CX_m_C-), α-conotoxins are divided into several subtypes, such as 3/5, 4/3, 4/4, 4/6, 4/7, and 5/5 subtypes (Lebbe et al., 2014; Giribaldi and Dutertre, 2018). They selectively inhibit the neuronal and muscle (nAChRs) or GABA_B_ receptor (GABA_B_R)-coupled N-type calcium (Ca_V_2.2) channel (Adams et al., 2012; Giribaldi and Dutertre, 2018). Several α-conotoxins, such as Vc1.1, RgIA, PeIA, and AuIB, have been shown to alleviate different types of chronic pain including chemotherapy-induced neuropathic pain (Satkunanathan et al., 2005; Klimis et al., 2011; Napier et al., 2012; Satkunanathan et al., 2012; Chhabra et al., 2014; Di Cesare Mannelli et al., 2014; Pacini et al., 2016). The analgesic activities of Vc1.1, RgIA, PeIA, and AuIB arise from their potent inhibition of the GABA_B_R-coupled Ca_V_2.2 channel (Daly et al., 2011; Adams et al., 2012; Chhabra et al., 2014; Di Cesare Mannelli et al., 2014). Besides α-conotoxins, ω-conotoxins, belonging to the O-superfamily of conotoxins, target N-type (Cav2.2) VGCCs and exhibit good efficacy in animal pain models (Lee et al., 2010; Jayamanne et al., 2013; Sousa et al., 2018; Chen et al., 2021; Yousuf et al., 2022). ω-Conotoxin MVIIA (SNX-111, ziconotide, Prialt®) was approved by the US FDA for the management of severe chronic pains in patients who were unresponsive to opioid therapy, and is now recommended as first-line IT monotherapy for cancer- and non-cancer-related pain (Deer et al., 2019). However, MVIIA has adverse side effects, including dizziness, nystagmus, somnolence, abnormal gait, and ataxia (Sanford, 2013).

In the present study, we report a novel 4/8 subtype α-conotoxin, Vt1.27 (NCCMFHTCPIDYSRFNC-NH_2_), identified from *Conus vitulinus* in the South China Sea by RACE methods. It was synthesized, and structurally and functionally characterized. Similar to other types of α-conotoxins, Vt1.27 has typical disulfide bridges (C^1^–C^3^, C^2^–C^4^) and displayed relatively weak activity at neuronal nAChR subtype α3β2 (IC_50_ = 1160 nM) expressed in *Xenopus* oocytes. However, Vt1.27 also inhibited N-type calcium (Cav2.2) channels expressed in HEK293T cells with an IC_50_ of 398 nM but did not inhibit voltage-gated sodium or potassium channels in DRG neurons. Furthermore, Vt1.27 exhibited potent anti-allodynic effect in rat partial sciatic nerve injury (PNL) and chronic constriction injury (CCI) pain models at the nmol/kg level with intramuscular injection, and higher than that of Vc1.1 in CCI rat models. To further probe the structure-activity relationship, Vt1.27 Alanine mutants were synthesized. The NMR structure of Vt1.27 was also determined and used to compare the Ca_V_2.2 binding mechanism of Vt1.27 and MVIIA by molecular dockings. These findings expand our knowledge of targets of α-conotoxins and provide a potent anti-allodynic peptide for the treatment of neuropathic pain.

## Materials and Methods

### Reagents and Animals

The pGEM-T Easy Vector System, *E. coli*. Dh5α, 2×Taq PCR Master Mix, and DNA Marker were obtained from TIANGEN Biotech Co., Ltd. (Beijing, China). The 3′-Full RACE Core Set Ver.2.0 kit was obtained from TaKaRa (Dalian, China). The Nucleic Acid purification kit was purchased from Dongsheng Biotech (Guangzhou, China). N-Fmoc-amino acids and HOBt were sourced from GL Biochem Ltd. (Shanghai, China). Rink resin was purchased from Tianjin Nankai Hecheng S&T Co. (Tianjin, China). *N,N′*-Dicyclohexylcarbodiimide (DCC) and Methanol were purchased from J&K Chemical Ltd. (Shanghai, China) and Honeywell Burdick & Jackson (Muskegon, MI United States), respectively. The other reagents were of analytical grade.

Adult male Sprague-Dawley rats (SD, 250 g, Beijing Animal Center, China) were housed in groups of eight and maintained on a 12 h light-dark cycle (light cycle from 8 am to 8 pm) at a temperature of 23 ± 2°C and relative humidity of 50%. Food pellets and water were available *ad libitum*. All experiments were conducted in accordance with the guidelines of the Beijing Institutes for Biological Sciences Animal Research Advisory Committee and conformed to the European Community directives for the care and use of laboratory animals.

### Cloning of α-Conotoxin Vt1.27

*Conus vitulinus* was harvested near Hainan Island in the South China Sea. The venom ducts were dissected from living cone snails and frozen immediately in liquid nitrogen. Total RNA was extracted and was reversed into cDNA as described previously (Liu et al., 2010). To amplify the cDNA encoding of the novel α-conotoxin, the following nested primers pairs were employed: the forward outer primer F1 (5′-ATG GGC ATG CGG ATG ATG TTC-3′) and inner primer F2 (5′-CTG TTG GTT GTC TTG GCA ACC AC-3′), designed based on the conserved sequence in the signal peptide of known αA-superfamily conotoxins and the reverse 3′-RACE outer primer R1 (5′-TAC CGT CGT CGT TCC ACT AGT GAT TT-3) and reverse 3′-RACE inner primer R2 (5′-CGC GGA TCC TCC ACT TGT GGT AGG G-3′), provided in the 3′-Full RACE Core Set Ver.2.0 kit. Both the first and the second PCR amplification reactions included an initial denaturation step carried out at 94°C for 4 min, followed by 30 cycles at 94°C for 30 s, then at 56°C for 30 s, and 72°C for 1 min, and a final extension step at 72°C for 10 min. After the final PCR products were analyzed on a 1% agarose gel, the appropriate bands were purified and the products were ligated into pGEM-T Easy Vectors and transformed into *E. coli.* DH5α cells. The positive clones containing the desired inserts were subsequently sequenced.

### Peptide Synthesis, Folding, and Disulfide Connectivity Analysis

Vt1.27 and its mutants were synthesized as described previously (Wang et al., 2015). Briefly, Vt1.27 was assembled and then cleaved from Rink resin by treatment with the cleavage solution (trifluoroacetic acid (TFA, 8.8 ml)/water (0.5 ml)/DTT (0.5 g)/Triisopropylsilane (0.2 ml). The released peptides were oxidized in 0.1M NH_4_HCO_3_ at room temperature, pH 8.0–8.2. The folding products were then purified by semi-preparative RP-HPLC. The final products were assessed by analytical reversed-phase HPLC.

The disulfide arrangement of Vt1.27 synthesized by the one-step oxidative folding was determined by comparison with peptide folding products with known disulfide connectivity (Ning et al., 2021). The linear peptide containing an acetamidomethyl (Acm) protecting group at the Cys^2^ and Cys^4^ position was folded by incubation in 0.1 M Tris-HCl (pH 8.0) at room temperature for 24–36 h. The folded products were further oxidized with an iodine buffer containing 30% CH_3_CN, 2% TFA, and 68% H_2_O for 10 min to form a peptide with the disulfide bridges “C^1^-C^3^, C^2^-C^4^”. This second oxidation product was co-applied with the one-step folding product of Vt1.27, and the disulfide connectivity was then determined.

### The Determination of NMR Solution Structure

Vt1.27 was dissolved in 500 μl of either 9:1 (v/v) H_2_O/D_2_O or 99.99% D_2_O (Cambridge Isotope Lab, MA, United States) with 0.01% trifluoroacetic acid (TFA, St. Louis, United States) at pH 3.0. The final peptide concentration was approximately 3.0 mM. NMR spectra were collected on Bruker Avance 400 and 600 MHz NMR spectrometers at 298K. The homonuclear DQF-COSY, TOCSY, and NOESY spectra were obtained in a phase-sensitive mode using time-proportional phase incrementation for quadrature detection in the t1 dimension. Pre-saturation during the relaxation delay period was used to suppress the solvent resonance unless specified otherwise. NOESY spectra were obtained with a mixing time of 300 ms. TOCSY spectra were obtained using the MLEV-17 pulse scheme for a spinlock of 120 ms. Each sample lyophilized from the hydrogen-containing solution was re-dissolved in a deuterium-containing solution in order to identify the slow exchange of backbone amide protons. All chemical shifts were referenced to the methyl resonance of 4,4-dimethyl-4-silapentane -1-sulfonic acid (DSS) used as the internal standard. The spectra were processed using Bruker Topspin 2.1 and analyzed by Sparky 3.1.

Distance constraints derived from the NOESY spectra of Vt1.27 were used for structural calculations using Cyana 2.1 software (Guntert, 2004). Dihedral angle restraints were determined based on the ^*3*^ *J*_*HN-Ha*_ coupling constants derived from the DQF-COSY spectral analysis. The *φ* angle constraints for some residues were set to −120 ± 40° for ^*3*^ *J*_*HN-Ha*_> 8.0 Hz and -65 ± 25° for ^*3*^ *J*_*HN-Ha*_< 5.5 Hz. In addition, backbone dihedral constraints were not applied for ^*3*^ *J*_*HN-Ha*_ values ranging from 5.5 to 8.0 Hz. The hydrogen bond constraints were added as target values of 2.2 Å and 3.2 Å for the NH(i)–O(j) and N(i)–O(j) bonds, respectively, based on the slow exchange of amide protons in hydrogen-deuterium exchange experiments.

According to the primary sequence, 100 random structures were generated to fit covalent and spatial requirements, and the 20 lowest energy conformers were submitted to a molecular dynamics refinement procedure using the Sander module of the Amber 9 program. The final outcomes were used for structural quality analysis using MOLMOL software, and the geometric qualities of the refined structures were evaluated using PROCHECK-NMR software. The data, including chemical shifts, were submitted to the BMRB database with access code 51264 for Vt1.27.

### Two-Electrode Voltage Clamp

Oocyte electrophysiology was carried out as described previously (Wang et al., 2015). α2, α3, α4, α7, β2, and β4 rat neuronal nAChR subunits were cloned into PNKS2 vectors. PGEMHE-α9, PSGEM-α10 and PNKS2 vector were a gift from Professors A.B. Elgoyhen and A. Nicke (Lörinczi et al., 2012; Boffi et al., 2013). *Xenopus laevis* oocytes were injected with 50.6 nl of RNase-free water containing 15–20 ng of cRNA (at a 1:1 M ratio) and maintained in ND96 solution (96 mM NaCl, 2.0 mM KCl, 1.8 mM CaCl_2_, 1.0 mM MgCl_2_.6H_2_O, 5 mM HEPES, pH 7.4) at 18°C. Electrophysiological recordings were performed 2–5 days post-injection using a two-electrode voltage clamp with an Axoclamp 900A amplifier (Molecular Devices, Sunnyvale, CA, United States). Electrodes were filled with 3M KCl and had a resistance of 0.5–2MΩ. Data acquisition was performed using a Digidata 1440A and pClamp 10.0 software (Molecular Devices). Data were analyzed using the ClampFit function in the pClamp 10.0 program. Oocytes were voltage clamped at −70 mV and gravity-perfused with ND96 solution at a rate of 1 ml/min. Acetylcholine (ACh)-evoked currents were elicited by 3 s pulses of gravity perfused agonist solution including 100 μM ACh. Membrane currents were filtered at 1 kHz and sampled at 10 kHz. All electrophysiological experiments were performed at room temperature (21–23°C). All data were presented as the mean ± SEM of measurements taken from 4-5 oocytes in each treatment group.

### Whole-Cell Recording From HEK293T Cells

Depolarization-activated calcium channel currents were recorded from HEK293T cells transiently expressing the human calcium channel subunits α_1B_, β_3_, and α_2_δ_1_ and green fluorescent protein (GFP). Cell transfection was obtained using either Lipofectamine 2000^®^ or calcium phosphate precipitation protocol (Liu et al., 2018), by using an equal ratio of calcium channel subunits, respectively 1:1:1. Whole-cell currents were recorded using a MultiClamp 700B Amplifier (Molecular Devices) and raw data were digitalized with a Digidata 1500 (Molecular Devices). Data were filtered at 1 kHz and sampled at 10 kHz by using pClamp 11. Cells were superfused by a gravity-driven system (AutoMate Scientific, Inc. Berkeley, CA) at a constant flow rate of 2 ml/min with an external solution containing (in mM): 110 NaCl, 10 BaCl_2_, 30 tetraethylammonium-Cl, 1 MgCl_2_, 5 CsCl, 10 D-glucose, 10 HEPES, pH adjusted to 7.3 with TEA-OH. Borosilicate fire-polished pipettes (1.5–3 MΩ) were backfilled with an internal solution containing (in mM): 125 K-gluconate, 2 MgCl_2_, 5 EGTA, 5 NaCl, 4 Mg-ATP, 10 HEPES, pH adjusted to 7.2 with KOH. HEK293T cells were depolarized from a holding potential of −80 mV for 20–200 ms to its peak current at 0 mV at a frequency of 0.2 Hz. Cell series resistance was compensated by ∼75% while leak and capacitive currents were subtracted online using a–P/4 pulse protocol. All experiments were carried out at room temperature (22–23°C) and for no longer than 72 h after transfection. *α-*Conotoxin Vt1.27 was prepared as 100 µM H_2_O stock solution and applied at a concentration of 1 μM, if not stated otherwise.

### Anti-allodynic Effect Test

Under pentobarbital (55 mg/kg) anesthesia, the classic rat partial sciatic nerve injury (PNL) model and chronic constriction injury (CCI) model were established as described previously (Bennett and Xie, 1988; Seltzer et al., 1990; Liu et al., 2018). All behavioral testing was performed 7 days after surgery. A successful PNL and CCI model were defined by a 30–50% decrease in mechanical paw withdrawal thresholds (PWT) for the right hind paw, in which the sciatic nerve in the thigh was injured (PNL) or ligated. Mechanical paw withdrawal thresholds were assessed using an Ugo Basile Analgesiometer (Ugo Basile, Italy).

For pharmacological testing, the rats were randomly assigned to five groups that each received one of the following treatments: saline (negative control), Vc1.1 (12 nmol/kg) (positive control), or Vt1.27. Vt1.27 and Vc1.1 were dissolved in 0.9% saline to a volume of 200 μl and administered ipsilaterally close to the injury site at a mid-thigh region in a volume of 200 μl. Three different doses of Vt1.27 (0.24, 1.6, and 12 nmol/kg) were administrated. Paw withdrawal thresholds in PNL and CCl models were measured at 2 and 4 h following i.m. administration. Data are expressed as mean pain threshold (g). One-way analysis of variance (ANOVA) was performed, and *p* < 0.05 was considered significant.

### Docking

The cryo-electron microscopy structures of Ca_V_2.2 and NMR solution structure of Eu1.6 were retrieved from Protein Data Bank (7VFS, 7VFU) and the BMRB database (access code 21060) (Liu et al., 2018; Dong et al., 2021; Gao et al., 2021). Vt1.27 and Eu1.6 were docked to the Ca_V_2.2 channel in the Hdock web server (http://Hdock.phys.hust.edu.cn/.). The top 10 docked conformations of the Vt1.27 and Eu1.6 were selected for further analysis. The optimum binding mode was finally determined based on the scoring and experimental data.

## Results

### Identification of a Novel 4/8 Subtype α-Conotoxin Vt1.27

A novel conotoxin precursor was cloned from *Conus vitulinus* by 3’ RACE using the conserved signal peptide sequence of the A-superfamily of conotoxins. The mature toxin sequence was predicted as NCCMFHTCPIDYSRFNC-NH_2_ with a cysteine pattern of CCX_4_CX_8_C, suggesting that this peptide belongs to the novel α4/8 family of conotoxins. Vt1.27 exhibits a much lower sequence similarity in the mature peptide region to other α-conotoxins except for the highly conserved Cys residues. The cDNA sequence has been submitted to GenBank (accession number HM211180).

### Peptide Synthesis and Characterization of Vt1.27 and Its Variants

**FIGURE 1:**
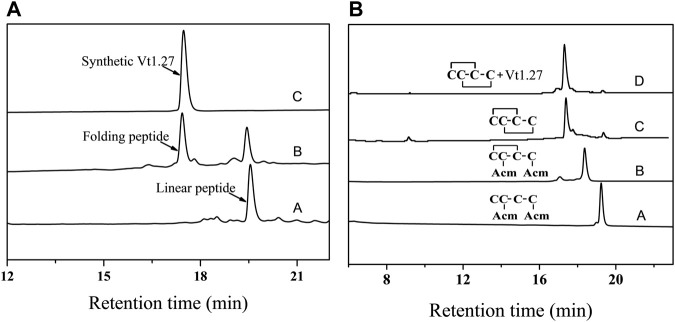
HPLC analyses of folded products of linear and Acm-modified Vt1.27. A. Folded products of linear Vt1.27: **(A)** Linear peptide; **(B)** One-step oxidized products; **(C)** Purified product of Vt1.27; B: **(A)** Linear peptide with Acm modifications at Cys2 and Cys 4; **(B)** First oxidized product; **(C)** Second oxidized product; **(D)** Co-elution of the two-step folding products and the purified product Vt1.27. Samples were applied to a Calesil ODS-100 C_18_ column (4.6 mm × 250 mm) and eluted by applying a 24 min linear gradient of 10–50% acetonitrile (0.1% TFA) at a flow rate of 1 ml/min, 214 nm.

In order to probe the target and functional groups, Vt1.27 and its alanine mutants or variants with basic residue Lys were synthesized. After the linear Vt1.27 was folded, two major peaks were observed (Figure 1A,B). Mass spectral analysis showed that the first peak is the correct folding product of the linear Vt1.27 with the expected molecular weight (Supplementary Figure S19). The final synthesized Vt1.27 was further assessed using analytical reversed-phase HPLC, and the results showed that its purity is >95%. The folding patterns of Vt1.27 mutants are similar to Vt1.27, and the mass spectra of pure products (Supplementary Figures S20–S33) are consistent with the theoretical molecular weight.

The disulfide connectivity of Vt1.27 was determined by comparing the folded peptide product with known disulfide connectivity. HPLC results of the one-step and two-step folding of the acetamidomethyl (Acm) protected linear peptides are shown in Figure 1B–D. The retention time of the synthesized Vt1.27 in the one-step folding is identical to that of Vt1.27 with the disulfide connectivity of “C^1^-C^3^, C^2^-C^4^” formed in the two-step oxidation process (Figure 1B–D), indicating the disulfide bond connectivity of Vt1.27 is “C^1^-C^3^, C^2^-C^4^”.

### The NMR Solution Structure of Vt1.27

**FIGURE 2:**
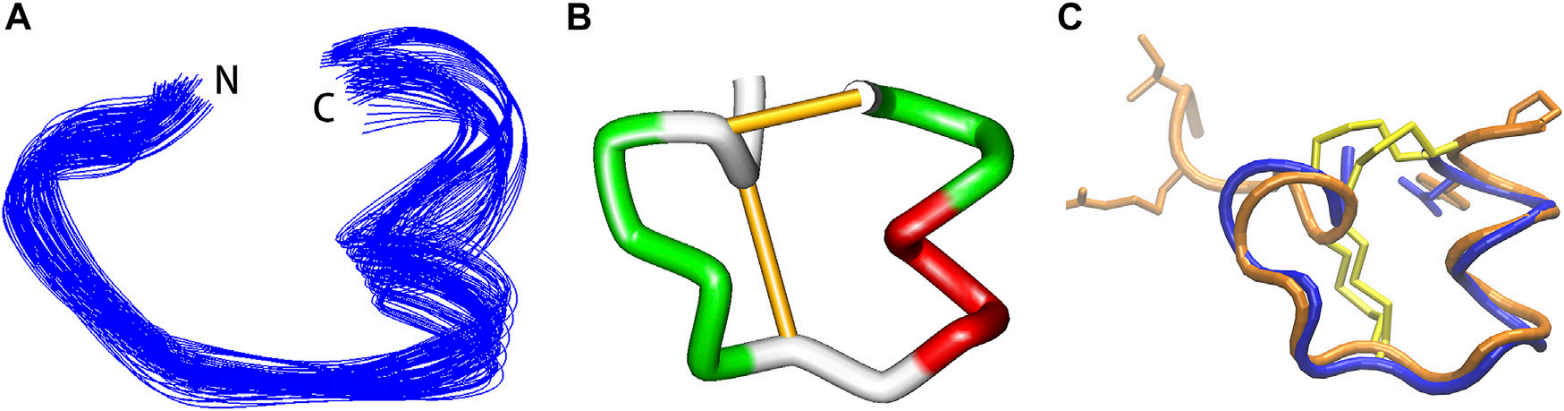
NMR structure of Vt1.27. **(A)** Backbone ensemble of 20 lowest energy structures. **(B)** Ribbon representation of the closest-to-mean structure. **(C)** Superimposition of Vt1.27 (blue) and Pl168 (orange). The side chains of Cys residues are shown in yellow. The side chains of different residues between Vt1.27 and Pl168 are also shown in **(C)**.

The structural statistics for Vt1.27 are given in Table 1. The ensemble of the 20-lowest energy structures is shown in Figure 2A and the ribbon representation is shown in Figure 2B. Vt1.27 showed a relatively incompact folding (Figure 2B). The 3_10_ helix was composed of residues Ile10 to Tyr12. An observed γ turn was composed of residues Phe5 to Thr7, as verified by the H-D exchange experiment. Another β turn was observed from residue Ser13 to Asn16.

**TABLE 1:** Structural statistics of the ensemble of 20 structures of Vt1.27 after CYANA calculation.

Residue	Vt1.27
Intra-residue	80
Sequential	47
Medium range	20
Long range	1
H bond constraints	4
Dihedral constraints	4
Cyana target function (Å)	0.12 ± 0.03
RMSD to mean coordinates	
Mean global backbone atoms RMSD	0.69 ± 0.17
Mean global heavy atoms RMSD	1.38 ± 0.29
Rachandran statistics from PROCHECK_NMR	
Most favored regions, %	55.4
Additional allowed regions, %	44.6
Generously allowed regions, %	0.0
Disallowed regions, %	0.0

### Activity of Vt1.27 and Mutants on Neuronal nAChRs

**FIGURE 3:**
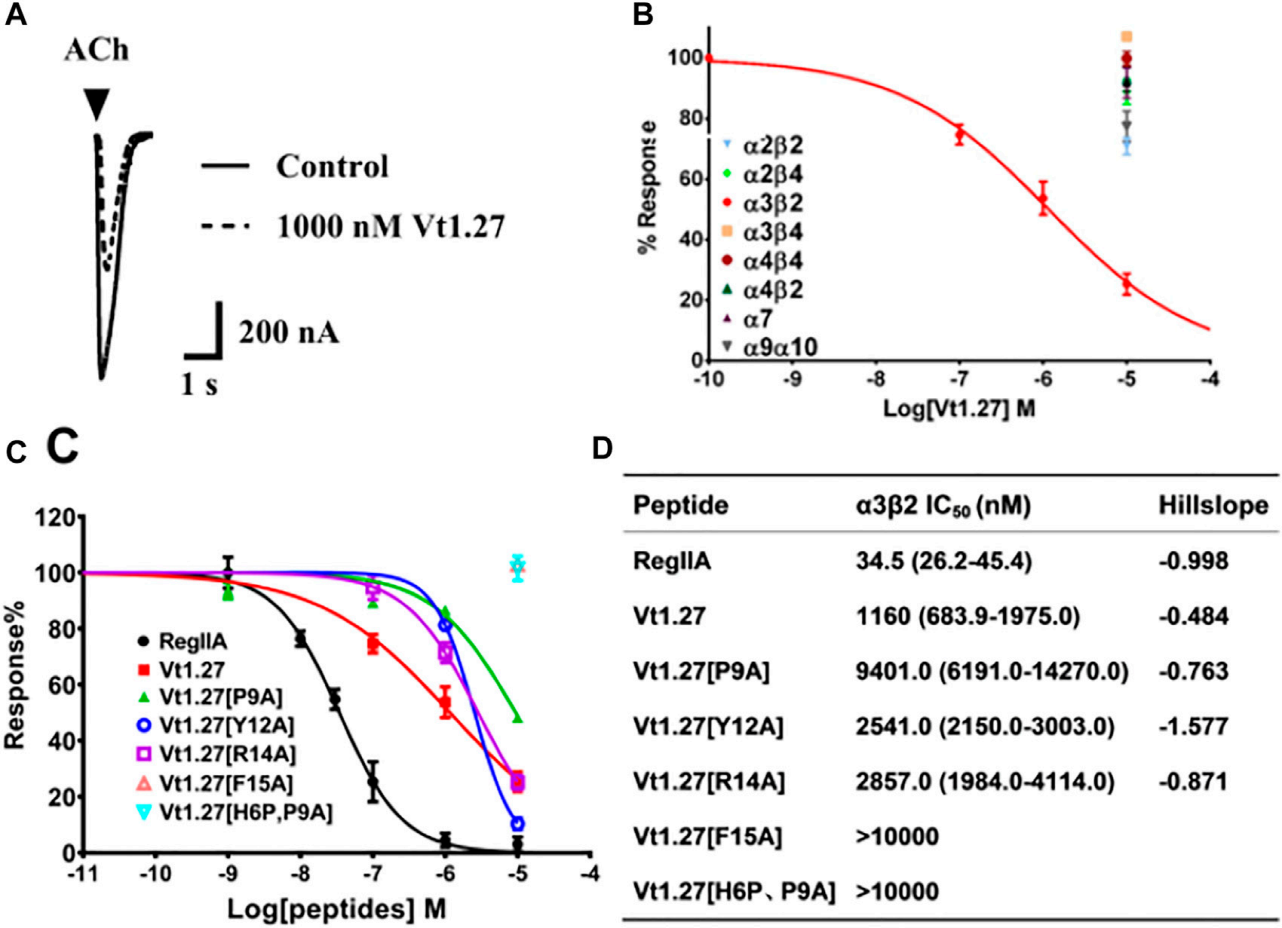
Activity of Vt1.27 and its variants at rat nAChR subtypes expressed in *Xenopus* oocytes. **(A)** Representative superimposed ACh-evoked currents recorded from oocytes expressing α3β2 nAChRs in the absence (control) and presence of Vt1.27. **(B)** Concentration-response relationships obtained for Vt1.27 inhibition of various rat nAChR subtypes. **(C)** Concentration-response curves for the inhibition of α3β2 nAChR by Vt1.27 variants. Peptides were applied by perfusion to oocytes expressing nAChRs as described in *Materials and Meth*ods. **(D)** Summary of IC_50_’s obtained for RegIIA, Vt1.27 and analogues at rat α3β2 nAChRs. Numbers in parentheses indicate 95% confidence intervals. *n* = 4–6 oocytes.

Vt1.27 was tested on various neuronal nAChRs expressed in *Xenopus* oocytes. As shown in Figure 3, Vt1.27 inhibited the rat neuronal α3β2 subtype with an IC_50_ of 1.16 μM, but 10 μM Vt1.27 exhibited minor inhibitory activity at rat α7, α3β4, α4β2, α2β2, α2β4, α9α10 and α3β4 nAChR subtypes (inhibition ratio <30%, Figure 3B). The replacement of Pro^9^, Tyr^12^, Arg^14^, Phe^15^ by Ala resulted in a decrease in the potency of Vt1.27 at the α3β2 nAChR subtype (Figure 3C,D), especially for the replacement of Pro^9^ by Ala with a sharp decrease in activity. The further modification of His^6^ in Vt1.27 [P^9^A] with Pro also led to a significant loss of potency.

### Activity of Vt1.27 and Mutants on Ca_V_2.2 Expressed in HEK293T Cells or DRG Neurons

**FIGURE 4:**
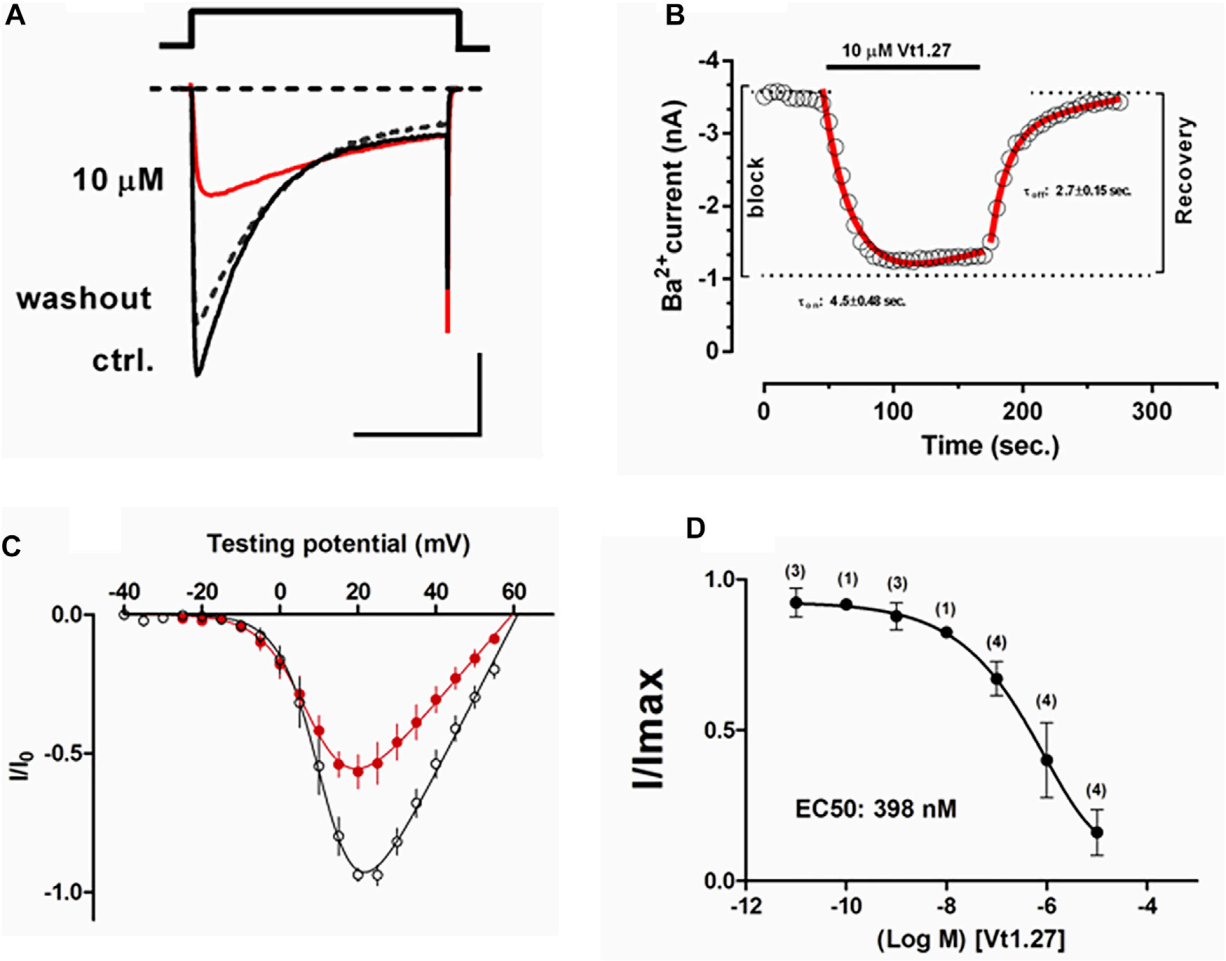
Effects of Vt1.27 on depolarization-activated Ba^2+^ currents in HEK293T cells. **(A)** Representative depolarization-activated whole-cell Ba^2+^ currents (black). Cells were pulsed from a holding potential HP of −80 mV for 200 ms at a frequency of 0.2 Hz Vt1.27 (10 µM) inhibition is indicated in red. The dashed line refers to the washout of Vt1.27. Scale: 1 nA, 100 ms. **(B)** Representative on and off kinetics upon application of 10 µM Vt1.27. The off kinetics suggests a full recovery of the current. **(C)** Current-voltage (I-V) relationship obtained in the absence and presence of 1 µM Vt1.27. **(D)** Concentration-response relationship obtained for Vt1.27 inhibition of Ca_V_2.2 (IC_50_ = 398 nM). Data shown in C and D represent the mean ± SEM (*n* = 7).

The effects of Vt1.27 on Ca_V_2.2 were determined using HEK293T cells stably expressing human Ca_V_2.2, α_2_δ_1,_ and β_3_. The results showed that 1 µM Vt1.27 inhibited Ba^2+^ currents in a voltage-dependent manner (Figure 4). Additional confirmation of block was obtained under various applied concentrations ranging from 10 pM to 10 µM. Fast on and off kinetics for Vt1.27 inhibition of 4.5 ± 0.48 s and 2.7 ± 0.15 s, respectively, was determined by a mono-exponential fitting function. In mouse DRG neurons, 1 μM Vt1.27 inhibited high voltage-activated (HVA) Ba^2+^ current (I_Ba_) amplitude by approximately 20% (Supplementary Figure S1). In addition, the application of the selective GABA_B_R antagonist, CGP 55845, failed to antagonize Vt1.27 inhibition of I_Ba_ in HEK293T cells co-transfected with GABA_B_R and Ca_V_2.2 (Supplementary Figure S2). Vt1.27 (5 μM) also did not significantly inhibit L-, or T-type calcium channels expressed in HEK293T and voltage-gated sodium or potassium channels in DRG neurons (Supplementary Figures S3–S4). These results demonstrate that Vt1.27 selectively inhibits Ca_V_2.2.

**FIGURE 5:**
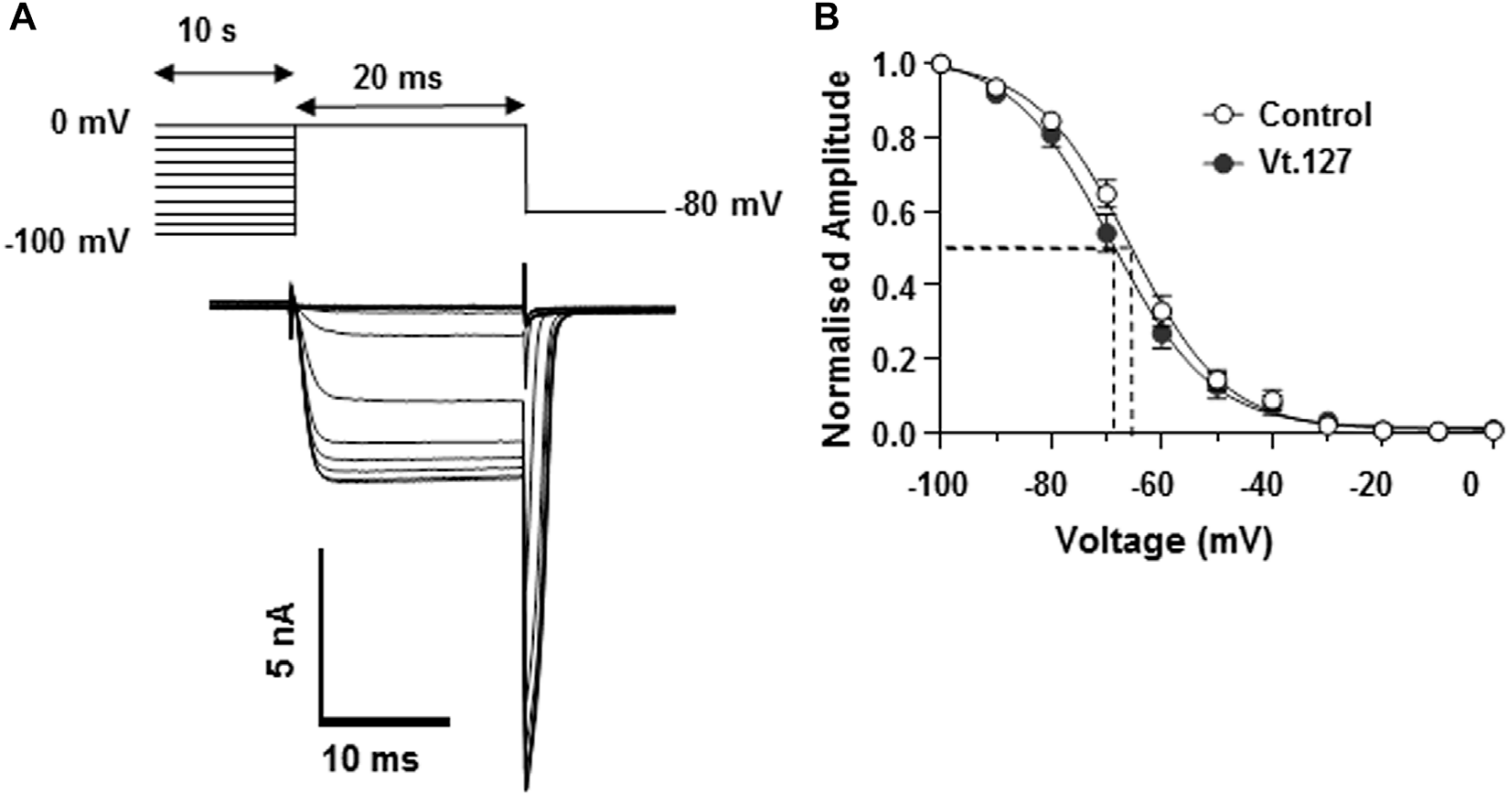
Effects of Vt1.27 on steady-state inactivation of Cav2.2 channels expressed in HEK293T cells. **(A)** Pre-pulse potentials (−100 to 0 mV in 10 mV increments for 10 s) were applied prior to a test pulse (20 ms) determined from the peak current and repeated every 15 s from a holding potential of −80 mV. Representative superimposed Ba^2+^ current traces obtained from different holding potentials. **(B)** Currents normalized to the maximum current amplitude plotted as a function of holding potential. Data points were fitted with a single Boltzmann function. Half-maximum inactivation (V_0.5, inact_) was shifted by 3 mV in the presence of 10 µM Vt1.27 (−69.0 ± 1.0 mV, *n* = 7, filled circles) compared to control (−66.0 ± 0.7 mV, *n* = 5, open circles).

The effects of Vt1.27 inhibition on steady-state inactivation (SSI) of Ca_V_2.2 channels expressed in HEK293T cells were also examined. A statistically significant shift (**p < 0.01) of half-maximum inactivation (V_0.5, inact_) for Vt1.27 (10 µM) was observed on Ca_V_2.2 channels (−69.0 ± 1.0 mV, n = 7) compared to control (−66.0 ± 0.7 mV, *n* = 5) (Figure 5).

**FIGURE 6:**
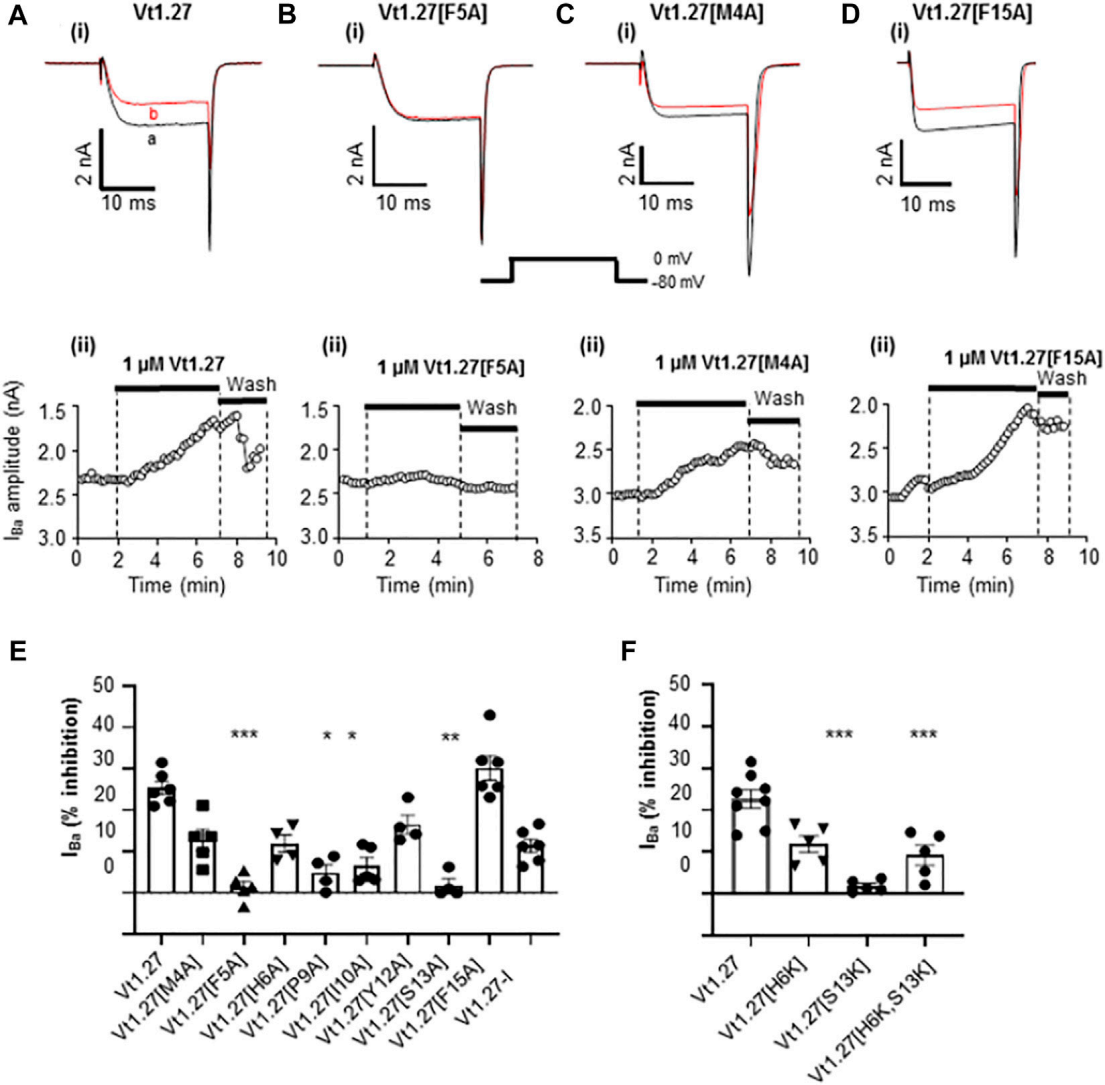
Effects of Vt1.27 alanine mutants on Ca_V_2.2 channels expressed in HEK293T cells. (A-Di) Representative depolarization-activated Ba^2+^ currents (I_Ba_) elicited from a holding potential of −80 mV to a test potential of 0 mV (20 ms duration; 0.2 Hz) from HEK293 cells expressing Ca_V_2.2 in the absence (control, a) and presence of 1 µM Vt1.27 analogues **(B)**. **(A–Dii)** Corresponding time plots of I_Ba_ amplitude before, during, and upon washout of peptide. **(E,F)** Bar graph showing mean percent inhibition of I_Ba_ at Ca_V_2.2 channels by different Vt1.27 mutant peptides. The columns are analyzed using the Kruskal-Wallis test and further by using Dunn’s multiple comparison test. Significant differences vs. the effect of wild-type Vt1.27 at *p < 0.05, **p < 0.01 and ***p < 0.001 (n = 4-6 per each column).

An alanine scan of Vt1.27 showed that replacement of Phe^5^ and Ser^13^ by Ala resulted in a complete loss of activity of Vt1.27 at Ca_V_2.2 whereas replacement of Met^4^, His^6^, Pro^9^, Ile^10^, and Thr^12^ by Ala caused a decrease in inhibitory activity (Figure 6). Replacement of Phe^15^ by Ala was equipotent to the native peptide Vt1.27. Further replacement of His^6^ with glycine resulted in no further change in inhibitory activity as caused by alanine replacement. However, the replacement of amino acid cassette Cys^3^-His^6^ along with Phe^15^ resulted in a complete loss of activity (Figure 6F).

### Effects of Acute Administration of Vt1.27 in Rat PNL and CCI Pain Models

**FIGURE 7:**
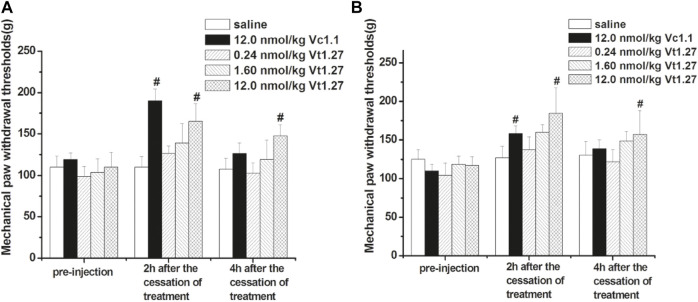
Anti-allodynic effects of Vt1.27 in rat PNL and CCI pain models. PNL **(A)** and CCI **(B)** rats (*n* = 8/group) treated with sterile saline (i.m.), Vc1.1 (12 nmol/kg, i.m.), and Vt1.27 (0.24, 1.6, and 12 nmol/kg, i.m.). The bar graph shows the mean pain threshold (g) pre-injection and at 2 h, 4 h following different treatments. The different groups were analyzed by one-way ANOVA followed by the LSD test for multiple comparison tests at the 0.05 level of significance. #*p* < 0.05 *vs*. Saline.

The anti-allodynic effect of Vt1.27 was determined in rat PNL pain models. At two hours after the ipsilateral muscular injection of different doses of Vt1.27 (0.24, 1.6, and 12 nmol/kg, i.m.) (Figure 7A), the pain threshold was 126.2 ± 9.16 g, 138.8 ± 23.6 g, and 165.0 ± 21.4 g, respectively, which are significantly higher than the initial pain threshold (98.8 ± 12.4, 103.8 ± 16.0, and 110.0 ± 17.8 g) with the increased mechanical pressure threshold of 27.4, 35.0, and 55.0 g. The increase in mechanical response threshold of Vt1.27 (12 nmol/kg) is slightly lower than for the same dose of α-conotoxin Vc1.1 (the elevation value of pain threshold was 55.0 and 71.2 g, respectively). However, at 4 h after peptide administration, the pain threshold of Vt1.27 (12 nmol/kg) is higher than the Vc1.1 group and the elevation value of the pain threshold was 37.6 g (Vt1.27) and 7.4 g (Vc1.1), respectively.

The anti-allodynic effect of Vt1.27 was further tested in the rat CCI pain model. Similar to the results of PNL experiments, the pain threshold significantly increased after administrating a different dose of Vt1.27 (Figure 7B). At 2 and 4 h after cessation of treatment, the high dose of Vt1.27 (12 nmol/kg) resulted in a significant increase in pain threshold (67.2 and 40.0 g), and the pain threshold elevation percentage (56.4 ± 14.9% and 33.3 ± 15.3%, n = 8) was higher than the Vc1.1 group (44.3 ± 9.1% and 26.1 ± 10.0%, n = 8). In addition, Vt1.27 did not affect locomotor activity even at a high dose (12 nmol/kg or higher).

## Discussion

Vt1.27 was the first 4/8 α-conotoxin cloned in 2010. Protein alignment revealed that Vt1.27 had no similarity in the mature peptide region to the other conotoxins except for Pl168 (Wilson et al., 2020) (Table 2). Unlike most α-conotoxins (Lebbe et al., 2014; Giribaldi and Dutertre, 2018). Vt1.27 has no conserved Pro residue in the first loop. In addition, Vt1.27 possesses many uncharged and hydrophobic amino residues and exhibits low water solubility. Although Vt1.27 represents high sequential identity with Pl168 in loop1 and loop2, which also has a 4/8 loop framework, it does not mean that Vt1.27 has the same structure as Pl168. The α-helix from Cys^6^ to Phe^9^ in Pl168 was not observed in Vt1.27, instead a γ turn composed of residues Phe^5^ to Thr^7^ was observed. Interestingly, the signal of amide protons of Thr^7^ in Vt1.27 and Thr^11^ in Pl168 were present in the H-D exchange experiment, indicating this residue was involved in secondary structures. The long α-helix from Ile^14^ to Tyr^20^ in Pl168 is divided into two parts in Vt1.27: one 3_10_ helix composed of residues Ile10 to Tyr^12^ and one β turn observed from residue Ser^13^ to Asn^16^ (Figure 2C). These differences may be caused by additional 4 residues in N-terminus, 1 residue in C-terminus and 1 residue in loop2 of Pl168.

**TABLE 2:** Amino acid sequences of Vt1.27 and other α-conotoxins.

Name	Origin	Amino Acid Sequences	Target
Vt1.27	*Conus vitulinus*	NCCMFHTCPIDYSRFNC^a^	α3β2 nAChR, Ca_V_2.2
Eu1.6	*Conus eburneus*	GCCSNPACMLKNPNLC^a^	Ca_V_2.2
Pl168	*Conus planorbis*	GIRGNCCMFHTCPIDYSRFYCP	Low activity at Ca_V_2.2
G1.9	*Conus geographus*	ECCKDPSCWVKVKDFQCPGASPPN	No activity at nAChRs

a carboxyl-terminal carboxamide.

To date, α-conotoxins largely target muscle or neuronal nAChR subtypes and a small number target Ca_V_2.2 channels via GABA_B_ receptor activation (Daly et al., 2011; Adams et al., 2012; Akondi et al., 2014; Chhabra et al., 2014; Di Cesare Mannelli et al., 2014; Cai et al., 2018). They belong to 3/5, 4/3, 4/4, 4/6, 4/7, and 5/5 subtypes, the only other 4/8 α-conotoxins reported are G1.9 and Pl168 mentioned above (Wilson et al., 2020; Tae et al., 2021). G1.9 (10 µM) is inactive at both muscle and neuronal nAChRs and Pl168 is inactive α7, α4β2, α3β2, or muscle-type nAChRs at peptide concentrations up to 100 µM. Application of 30 µM Pl168 only displayed a small (18%) inhibition of N-type (Ca_V_2.2) calcium channels in SH-SY5Y human neuroblastoma cells (Wilson et al., 2020). However, Vt1.27 potently inhibited both Ca_V_2.2 and nAChRs. The difference of Vt1.27 and Pl168 in pharmacology may be derived from the differences in N- or C-terminus and 1 residues in loop2, which results in the differences in solution structures.

The high anti-allodynic effect of Vt1.27 may be attributed to the inhibition of Ca_V_2.2 because it only displays a modest potency at α3β2 (IC_50_ = 1.2 µM) nAChRs although this receptor is distributed in the spinal cord and involved in pain (Napier et al., 2012). Our further structure-activity studies showed that Pro^9^ and Phe^15^ were the functional residues for its binding activity at α3β2 nAChR (Figure 3), and Tyr^12^ and Arg^14^ are relatively important for this target.

**FIGURE 8:**
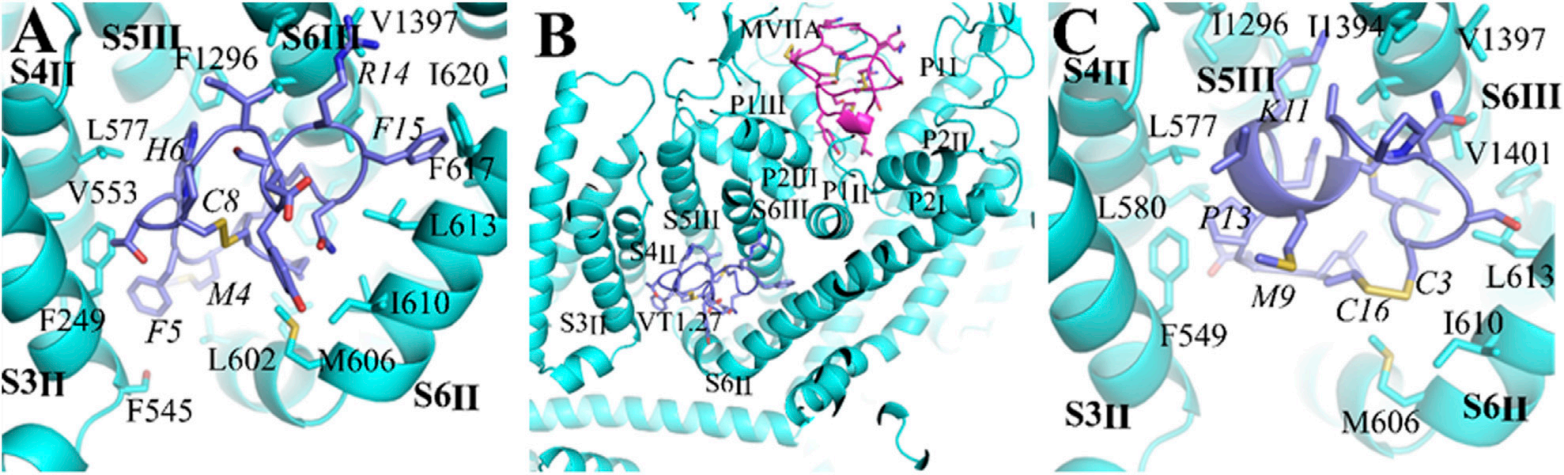
Comparison of binding modes of the Ca_V_2.2 channel with Vt1.27, MVIIA and Eu1.6. **(A) (C)** The interaction of α-conopetides Vt1.27 (purple) and Eu1.6 (purple), **(B)** General view of the interaction sites of Vt1.27 and a pore-blocker ω-conotoxin MVIIA (pink). The Ca_V_2.2 channel is shown in blue, the residues from Vt1.27 and Eu1.6 are labeled in italic format.

Recently, we reported a novel α-conotoxin Eu1.6 can target the Ca_V_2.2 channel directly (Liu et al., 2018). In addition, a number of ω-conotoxins also inhibit Ca_V_2.2 (Adams and Berecki, 2013). Compared to Eu1.6 or ω-conotoxins (IC_50_ = 0.1–0.2 μM), such as MVIIA and Bu8 (Chen et al., 2021), Vt1.27 exhibits a lower inhibitory activity at Ca_V_2.2 channels. However, Vt1.27 has moderate activity at α3β2 nAChRs, whereas Eu1.6 or ω-conotoxins have no apparent activity on neuronal nAChRs at 10 μM. To further understand the interactions between Vt1.27 and Ca_V_2.2, molecular dockings were performed in Hdock web server (http://Hdock.phys.hust.edu.cn/.) using a hybrid strategy of template-based modeling and ab initio template-free docking for protein–protein docking (Yan, et al., 2020). Ca_V_2.2 has four transmembrane domains (DI–DIV), each domain consists of six helices (S1-S6), and S1-S4 helices form the voltage-sensing domain (VSD). Four VSDs (VSD_I_–VSD_IV_) encircle the central pore formed by S5 and S6 helices from all four domains (Dong et al., 2021; Gao et al., 2021). According to the cryo-electron microscopy structures of human Ca_V_2.2, a hydrophobic pocket exists in the VSD of Ca_V_2.2, which is composed of S5_III_, S6_III_, S4_II_, S3_II,_ and S6_II_. Docking results show that the top 10 ranked conformations of Vt1.27 can embed in the pocket. Further analyses of the binding pocket of Vt1.27 (Figure 8A) show that the interactions of Ca_V_2.2 with Vt1.27 are primarily mediated by extensive hydrophobic interactions and van der Waals. The amino acid residues of Ca_V_2.2 within 4Å around Vt1.27 are shown in Figure 8A. The benzene ring of F15 is stabilized by interactions with hydrophobic residues from surrounding helices, such as L613, L616, F617, and I620, the distances are 2.8, 3.5, 4.0, and 3.7 Å, respectively. The strong hydrophobic interactions between M4 and V599 (2.9 Å), as well as T7 and F549 (1.7 Å), also exist. These binding modes are significantly different from that of MVIIA, which blocks the selectivity filter of Ca_V_2.2 (Dong et al., 2021; Gao et al., 2021) (Figure 8B, top). However, the binding modes of Vt1.27 and Eu1.6 with Ca_V_2.2 are similar, and Eu1.6 is also bound into the pocket. P13 is stabilized by contacts with adjacent hydrophobic residues, such as F549, L580, and L577, the distances are 2.2Å, 4.2Å, and 3.9Å, respectively. The other amino acid residues of Ca_V_2.2 in the range of 4 Å around Eu1.6 are shown in Figure 8C.

In summary, we have identified a novel 4/8 type α-conotoxin, Vt1.27, and shown that it inhibits both N-type calcium (Ca_V_2.2) channels and neuronal α3β2 nAChRs subtype. Its binding modes with Ca_V_2.2 are significantly different from that of ω-conotoxin MVIIA. Furthermore, Vt1.27 exhibits potent anti-allodynic effect in the rat partial sciatic nerve injury (PNL) and chronic constriction injury (CCI) models at nmol/kg levels after intramuscular (i.m.) administration. These findings expand our knowledge of targets of α-conotoxins and provide a potent anti-allodynic peptide analgesic for neuropathic pain.

## Data Availability

The datasets presented in this study can be found in online repositories. The names of the repository/repositories and accession number(s) can be found in the article/Supplementary Material.
