# Supplementary material for: A 4/8 Subtype α-Conotoxin Vt1.27 Inhibits N-Type Calcium Channels With Potent Anti-Allodynic Effect
Source: Front Pharmacol. 2022 Apr 29;13:881732. doi: 10.3389/fphar.2022.881732 (PMC9230573; doi:10.3389/fphar.2022.881732)
Supplement: Supplementary file 1 [file DataSheet1.DOC]

**Supplementary Material**

**A Novel 4/8 Subtype α-Conotoxin Vt1.27 Inhibits N-type Calcium Channels and Displays Potent Analgesic Activity**

Contents……………………….….…………..……………………….….…Page

1. Methods

1.1 Electrophysiological recording of whole-cell Ba^2+^ currents in mouse DRG neurons..……………………………………………..………………..……………2

1.2 Electrophysiological recording of L-(Cav1.2) and T-(Cav3.2) type calcium channels.……………………………………….……………………………………2

1.3 Electrophysiological recording of Na^+^ and K^+^ currents in rat DRG neurons….…3

Supplementary Figure 1. Vt1.27 inhibition of high voltage-activated Ba^2+^ current in mouse DRG neurons. ….….……………………….. ….……………………….4

Supplementary Figure 2. Effect of 1 µM Vt1.27 on HEK293T cells co-transfected with human GABA_B_ receptor R1 + R2 subunits and Cav2.2 channels……………..5

Supplementary Figure 3. Effect of Vt1.27 on voltage-gated Na^+^ and K^+^ currents in rat DRG neurons..…………………………………..………. ………………….….5

Supplementary Figure 4. Effect of Vt1.27 on L-(Ca_V_1.2) and T-(Ca_V_3.2) type calcium channels..………………………..……………………..……………………………6

Supplementary Figure 5. HPLC analysis of Vt1.27……………………..………..7

Supplementary Figure 6. HPLC analysis of Vt1.27[N1A]……………….….…8

Supplementary Figure 7. HPLC analysis of Vt1.27[F5A]……………………...9

Supplementary Figure 8. HPLC analysis of Vt1.27][H6A]………...………10

Supplementary Figure 9. HPLC analysis of Vt1.27[T7A]……………...…..11

Supplementary Figure 10. HPLC analysis of Vt1.27[P9A]………………...…..12

Supplementary Figure 11. HPLC analysis of Vt1.27[I10A]……………..…..13

Supplementary Figure 12. HPLC analysis of Vt1.27[D11A]………………...…14

Supplementary Figure 13. HPLC analysis of Vt1.27[Y12A]……………......….15

Supplementary Figure 14. HPLC analysis of Vt1.27[S13A]……………………16

Supplementary Figure 15. HPLC analysis of Vt1.27[S13K] …………………17

Supplementary Figure 16. HPLC analysis of Vt1.27[H6K,S13K]……………18

Supplementary Figure 17. HPLC analysis of Vt1.27[R14A]…………......…..19

Supplementary Figure 18. HPLC analysis of Vt1.27[F15A]……………......….20

Supplementary Figure 19. Mass spectrometry of Vt1.27………………….…....21

Supplementary Figure 20. Mass spectrometry of Vt1.27[N1A]………………..21

Supplementary Figure 21. Mass spectrometry of Vt1.27[M4A]………………..22

Supplementary Figure 22. Mass spectrometry of Vt1.27[F5A]…………….…..22

Supplementary Figure 23. Mass spectrometry of Vt1.27[H6A]……………..23

Supplementary Figure 24. Mass spectrometry of Vt1.27[T7A]…………..….…23

Supplementary Figure 25. Mass spectrometry of Vt1.27[P9A]……………...…24

Supplementary Figure 26. Mass spectrometry of Vt1.27[I10A]……………..…24

Supplementary Figure 27. Mass spectrometry of Vt1.27[D11A]……….…..25

Supplementary Figure 28. Mass spectrometry of Vt1.27[Y12A]………….…..25

Supplementary Figure 29. Mass spectrometry of Vt1.27[S13A]………….……26

Supplementary Figure 30. Mass spectrometry of Vt1.27[S13K]……….……26

Supplementary Figure 31. Mass spectrometry of Vt1.27[H6K,S13K]…….……27

Supplementary Figure 32. Mass spectrometry of Vt1.27[R14A]………………27

Supplementary Figure 33. Mass spectrometry of Vt1.27[F15A]……………….28

**1.1 Electrophysiological recording of whole-cell Ba^2+^ currents in mouse DRG neurons**

DRG ganglia were collected from C57BL/6 mice following all guidelines and regulations of the Animal Ethics Committee (AE16/10r19) of the University of Wollongong (UOW). Briefly, ganglia were dissected from thoracic and upper lumber regions after euthanizing mice with isoflurane inhalation and subsequent decapitation. They were then collected in ice-cold (4^o^C) Hanks Buffered Saline Solution (HBSS), free of Ca^2+^ and Mg^2+^, trimmed and transferred to a petri dish containing enzyme mix of Collagenase type II (3 mg/ml; Cat # LS004177, Worthington Biomedical Corp., Lakewood, NJ, USA) and Dispase (4 mg/ml; Cat # 17105041, GIBCO, Australia) in HBSS for digestion. After incubating this enzyme mix for 40 min at 37°C in 5% CO_2_ in a humidified incubator, the ganglia were washed 3-4 times using warm (37^o^C) F12/Glutamax (Cat # 31765-035, Thermo Fisher Scientific, Australia) media supplemented with heat-inactivated 10% foetal bovine serum (FBS; Cat # 10082-139, Thermo Fisher Scientific) and 1% penicillin/streptomycin (Pen/Strep; Cat # 15240-012, Thermo Fisher Scientific). The ganglia were then dissociated and triturated using progressively smaller fire-polished glass Pasteur pipettes and filtered through a 160 μm nylon mesh (Millipore Australia Pty Ltd, North Ryde, NSW) to remove undigested material. Neurons were then plated onto poly-D-lysine coated 12 mm glass coverslips (Sigma-Aldrich, Australia) allowed 3-4 hrs to adhere at 37°C. Finally, 1 mL of media was added to supplement the neurons, left overnight in the incubator, and transferred to a 30°C incubator to be used within 12-24 hrs.

**1.2 Electrophysiological recording of L-(Cav1.2) and T-(Cav3.2) type calcium channels**

Human embryonic kidney (HEK) 293 cells were cultured in modified Eagle’s medium (DMEM, Gibco) supplemented with 10% fetal bovine serum (FBS, Gibco) 100 units of penicillin, 100 units of streptomycin at 37℃ incubator with 5% CO_2_. Cells were transferred to a 24-well plate before transfection and then transfected with the cDNA(Cav1.2(α_1B_), Cav3.2(α_1A_)) for 4 h and the transiently transfected using lipofectamine2000 (Invitrogen) according to the manufacturer’s protocol.

Whole-cell voltage clamp recordings were performed 24–48 h after transfection as described previously (Liu et al., 2013; Tang et al., 2014) The patch pipettes were pulled from borosilicate glass capillaries with resistance of 2–4 MΩ in HEK293 cell experiments when filled with pipette solution. For calcium channel experiments, the extracellular solution contained (in mM): 135 N-methyl-D-glucamine (NMDG), 20 BaCl_2_^.^2 H_2_O, 2 MgCl_2_^.^6 H_2_O, and 10 HEPES, pH ~7.4. The intracellular solution contained (in mM): 135 CsCl, 10 NaCl, 10 HEPES, 5 EGTA, and pH was adjusted to 7.2 with CsOH. The experiments were recorded using an EPC-10 USB amplifier (HEKA, Lambrecht, Germany) and the currents were typically digitized at 1 kHz (for calcium channels) or 10 kHz (Nav1.4 and Nav1.5). Compensation of series resistance was 65–85% and all the currents were filtered at 3 or 5 kHz. All experiments were performed at room temperature (20–24 ºC).

**1.3 Electrophysiological recording of Na^+^ and K^+^ currents in rat DRG neurons**

DRG neurons with large diameter (>35 µm) and those with relatively small diameter (<20 µm) were chosen for measuring Na^+^ and K^+^ currents, respectively. Whole-cell voltage clamp recordings of voltage-gated ion currents were made in rat DRG neurons as described previously (Xiao, et al., 2003).

**REFERENCES**

Liu, R., Zhang, Z., Liu, H., Hou, P., Lang, J., Wang, S., Yan, H., Li, P., Huang, Z., Wu, H., Rong, M., Huang, J., Wang, H., Lv, L., Qiu, M., Ding, J., Lai, R. (2013). Human β-defensin 2 is a novel opener of Ca^2+^-activated potassium channels and induces vasodilation and hypotension in monkeys. Hypertension 62(2), 415–425. doi: 10.1161/HYPERTENSIONAHA.111.01076

Tang, C., Zhou, X., Huang, Y., Zhang, Y., Hu, Z., Wang, M., Chen, P., Liu, Z., Liang, S. (2014). The tarantula toxin jingzhaotoxin-XI (κ-theraphotoxin-Cj1a) regulates the activation and inactivation of the voltage-gated sodium channel Nav1.5, Toxicon 92, 6–13. doi: 10.1016/j.toxicon.2014.09.002

Xiao, Y.C., and Liang, S.P. (2003). Inhibition of sodium channels in rat dorsal root ganglion neurons by Hainantoxin-IV, a novel spider toxin. Sheng Wu Hua Xue Yu Sheng Wu Wu Li Xue Bao (Shanghai) 35(1), 82–85.


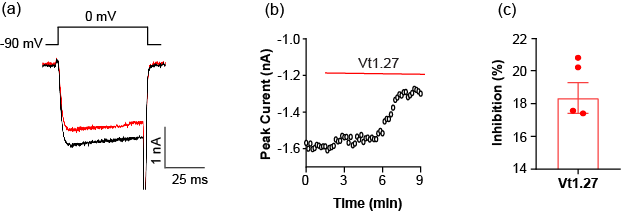


**Supplementary Figure 1.** Vt1.27 inhibition of high voltage-activated Ba^2+^ current (I_Ba_) in mouse DRG neurons. **(a)** Whole-cell I_Ba_ current recorded in the absence (control, black) and presence (red) of 1 µM Vt1.27 in mouse DRG neurons elicited by the voltage protocol shown above. **(b)** Representative time course of the effect of Vt1.27 on HVA I_Ba_ amplitude. Peptide was applied during the periods indicated by the red bar. **(c)** Bar graph of the percentage inhibition of HVA I_Ba_ amplitude (19 ± 0.9 %, n =4) in mouse DRG neurons. Data represent mean ± SEM and number of experiments presented within the bar.


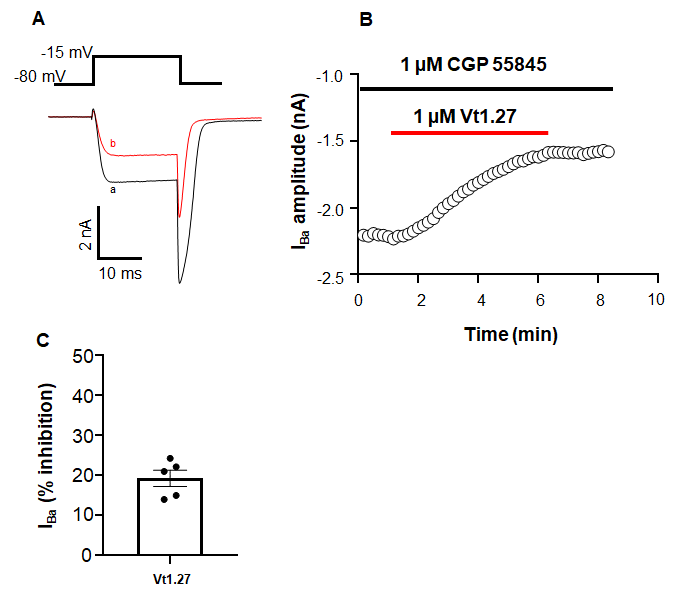


**Supplementary Figure 2.** Effect of Vt1.27 on HEK293T cells co-transfected with human GABA_B_ receptor R1 + R2 subunits and Cav2.2 channels. (**A**) Representative depolarization-activated Ba^2+^ currents (I_Ba_) elicited from a holding potential of −80 mV to a test potential of −15 mV (20 ms duration; 0.2 Hz) in the presence of the selective GABA_B_R antagonist, CGP 55845 (1 µM) alone (a) and co-applied with 1 µM Vt1.27 (b). (**B**) Corresponding time plot of I_Ba_ amplitude before and during application of 1 µM Vt1.27. (**C**) Bar graph showing percent inhibition of I_Ba_ at GABA_B_R-coupled Cav2.2 channels by Vt1.27 (19 ± 2%, n = 5). Data represent mean ± SEM and number of experiments presented within the bar.


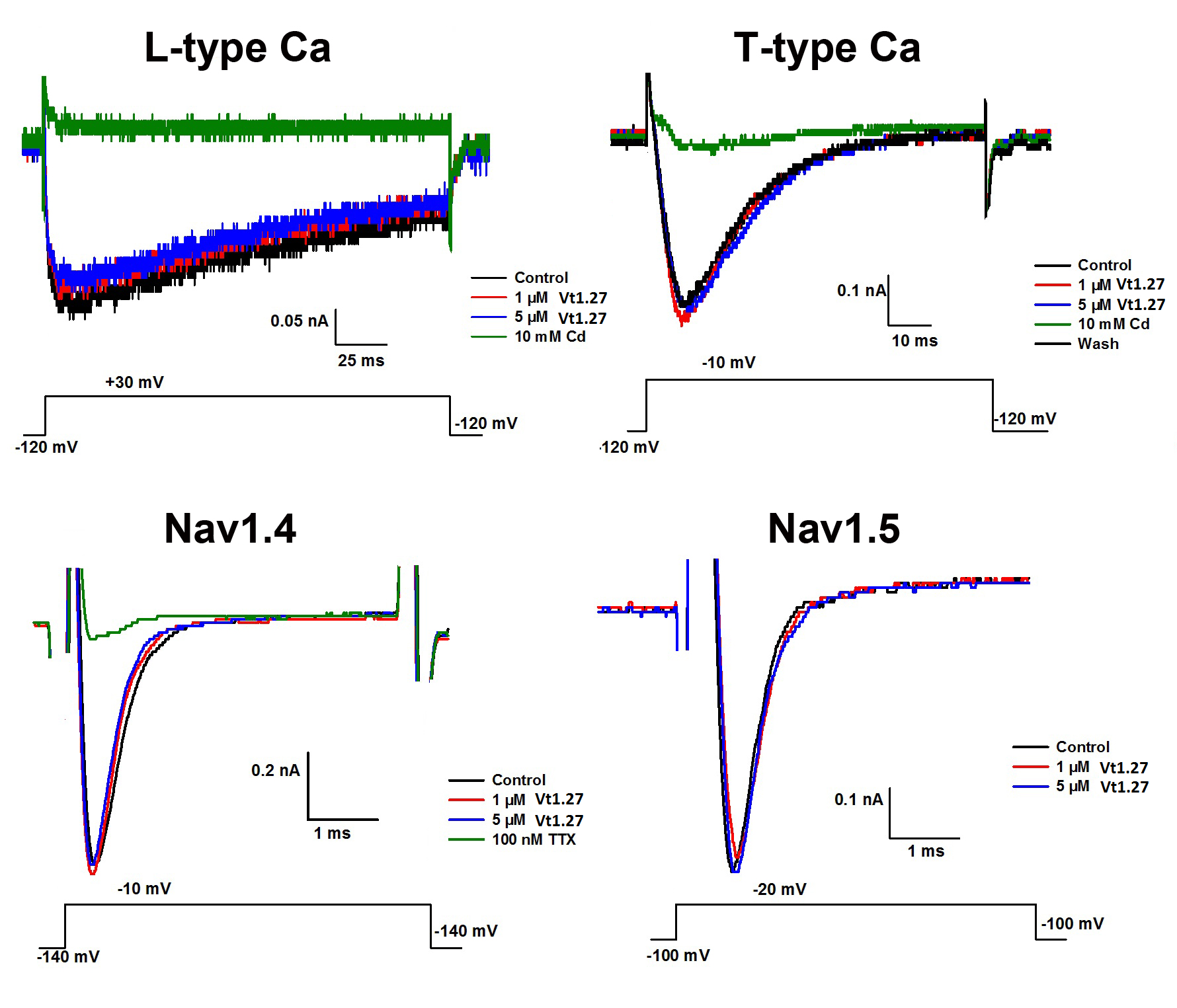


**Supplementary Figure 3**. Effects of Vt1.27 (1, 5 μM) on L- (Cav1.3) and T-(Cav3.2) type calcium channels expressed in HEK293 cells (n = 4-6 for each).


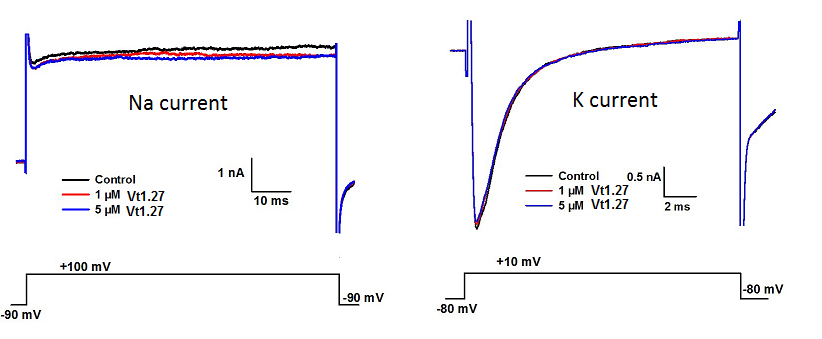


**Supplementary Figure 4.** Effect of Vt1.27 (1, 5 μM) on voltage-gated Na^+^ and K^+^ currents in rat DRG neurons (n = 4-6 for each).


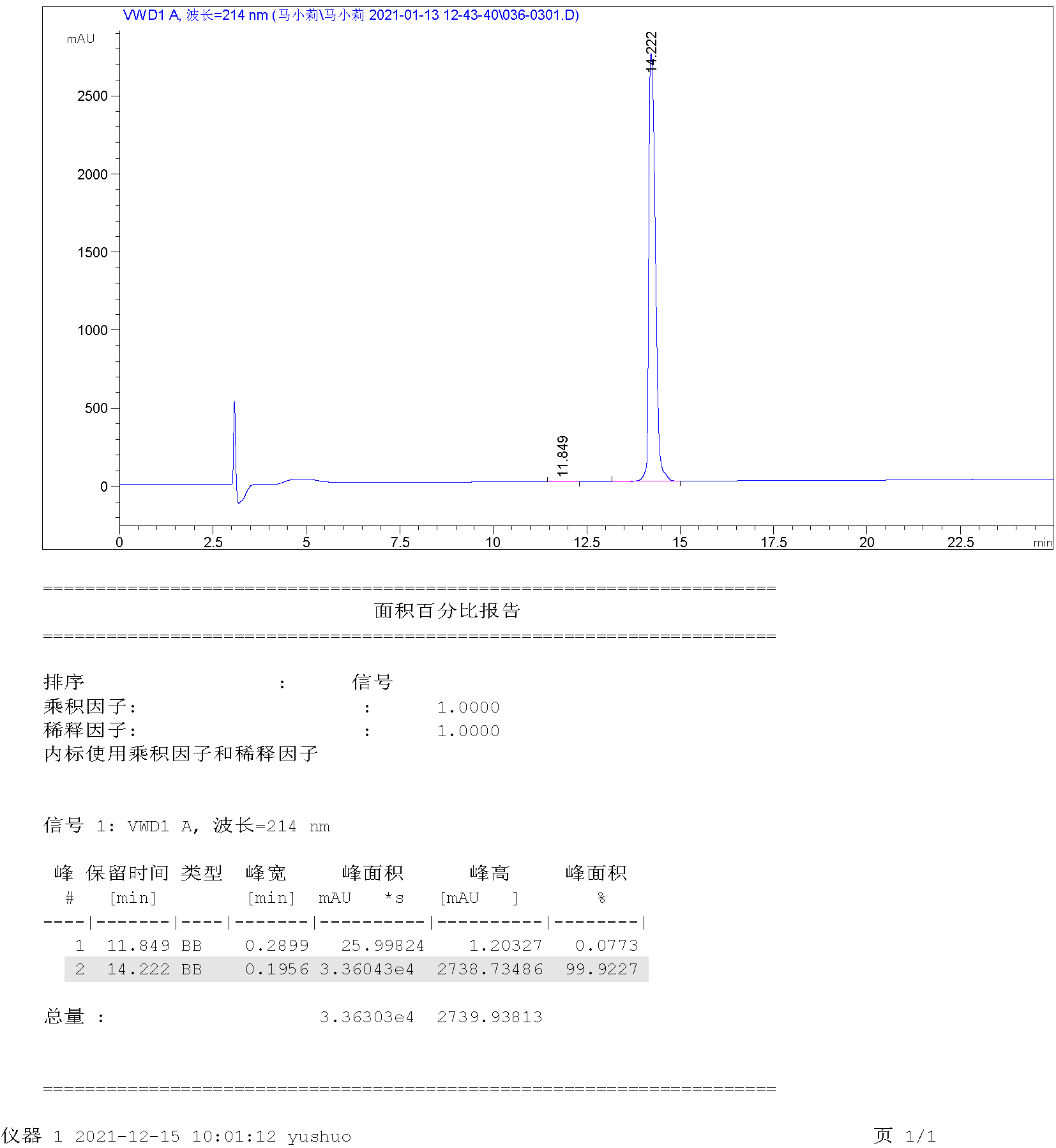


**Supplementary Figure 5.** HPLC analysis of Vt1.27 Samples were applied to an Agilent Eclipse Plus C18 (5 μm, 4.6 mm×250 mm) and eluted with a linear gradient of 0~10% B for 0-1 min; 10~50% B (B is acetonitrile containing 0.1% TFA) for 1-25 min. Absorbance was monitored at 230 nm. Flow rate was 1.0 ml/min.


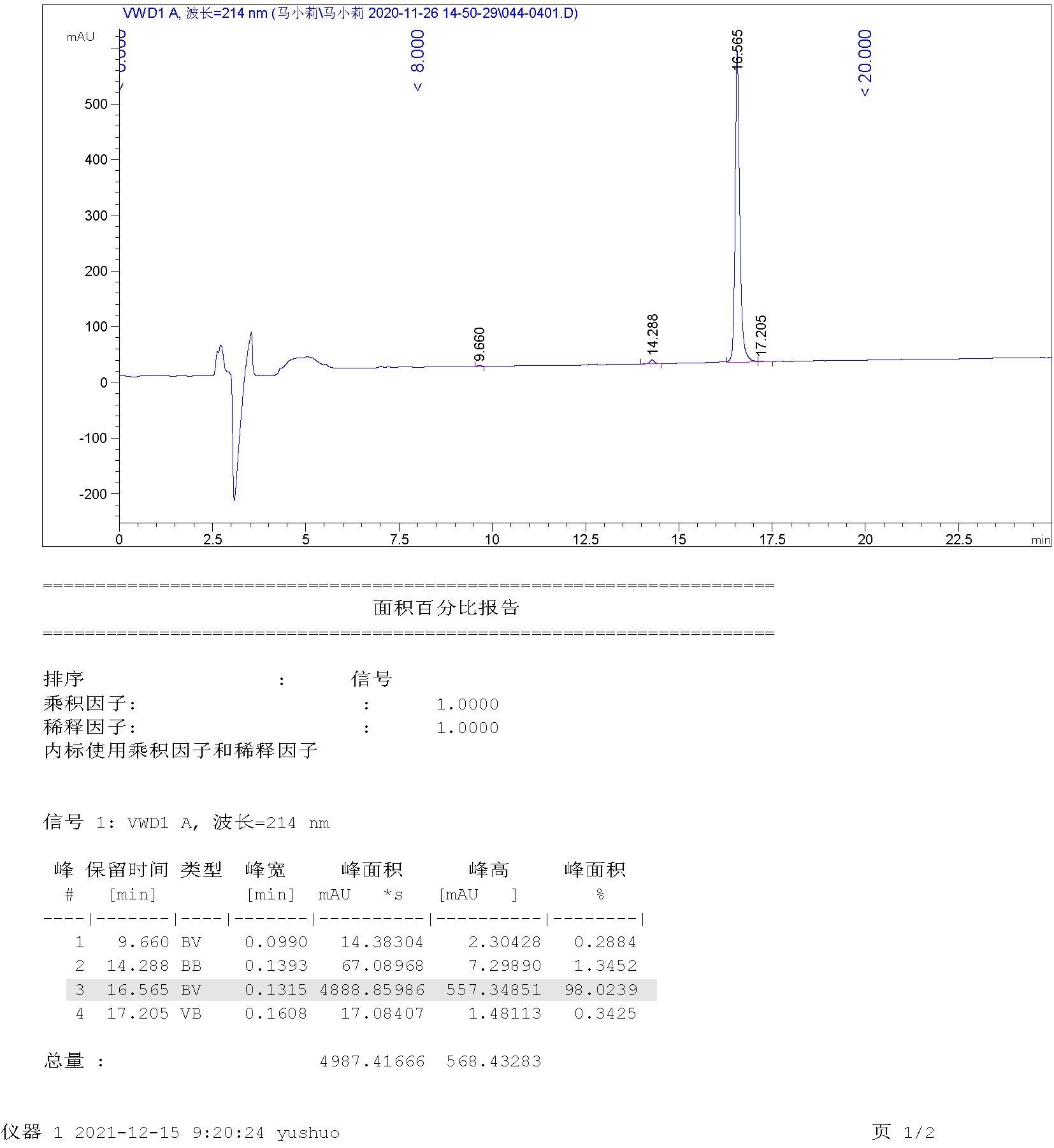


**Supplementary Figure 6.** HPLC analysis of Vt1.27[N1A]. Analytical conditions were the same as those described in Supplementary Figure 5.


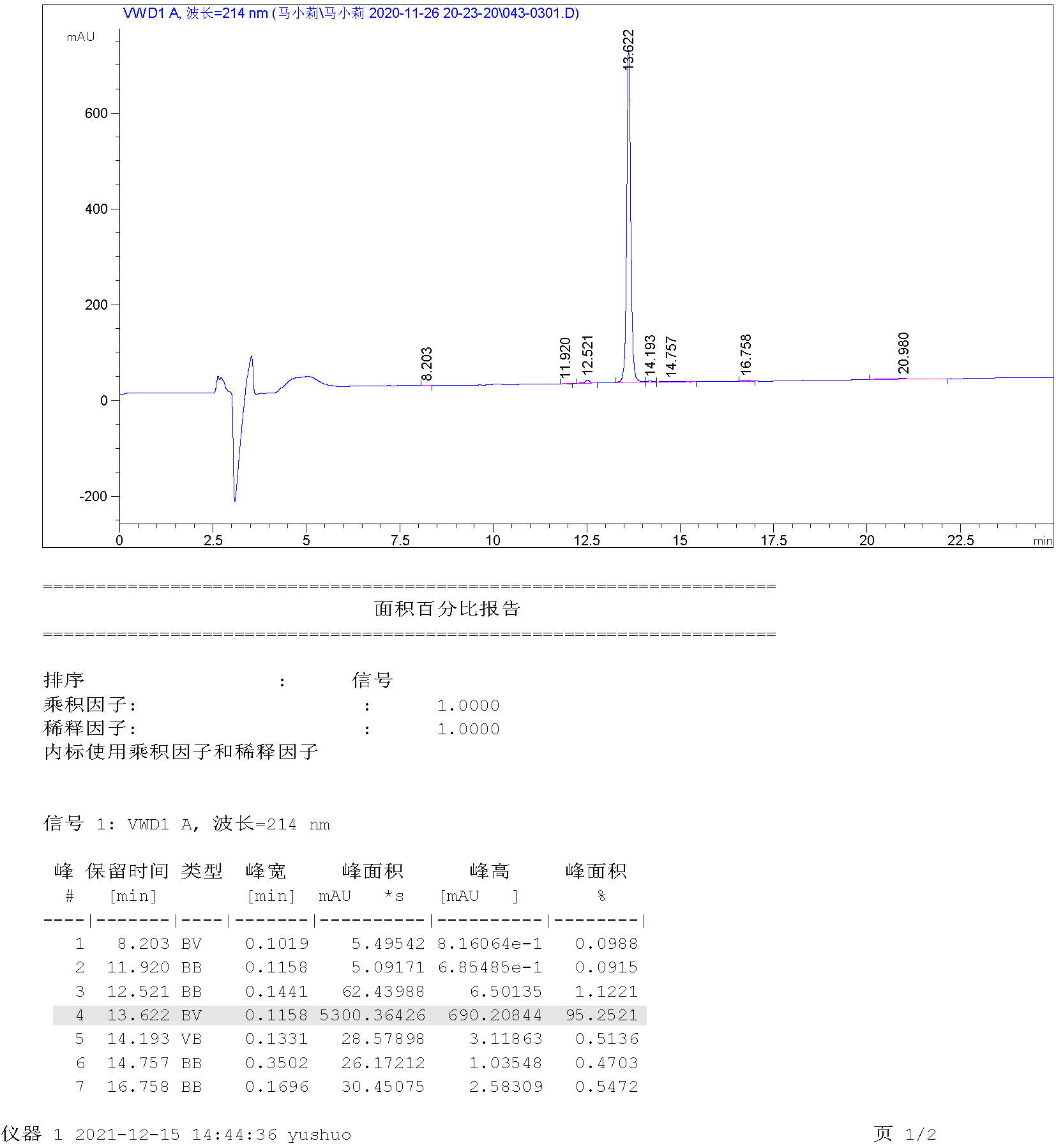


**Supplementary Figure 7.** HPLC analysis of Vt1.27[F5A]. Analytical conditions were the same as those described in Supplementary Figure 5.


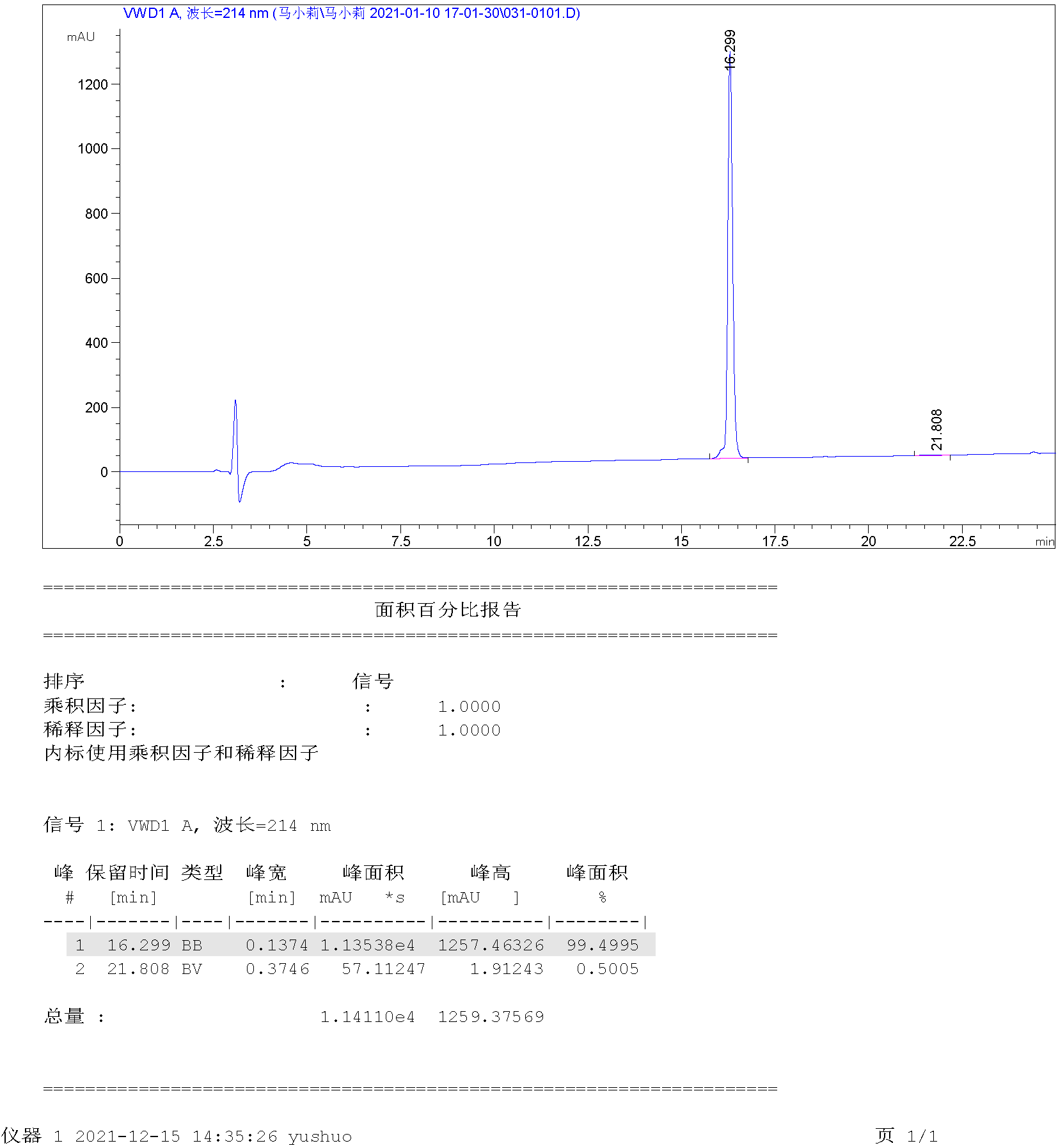


**Supplementary Figure 8.** HPLC analysis of Vt1.27[H6A]. Analytical conditions were the same as those described in Supplementary Figure 5.


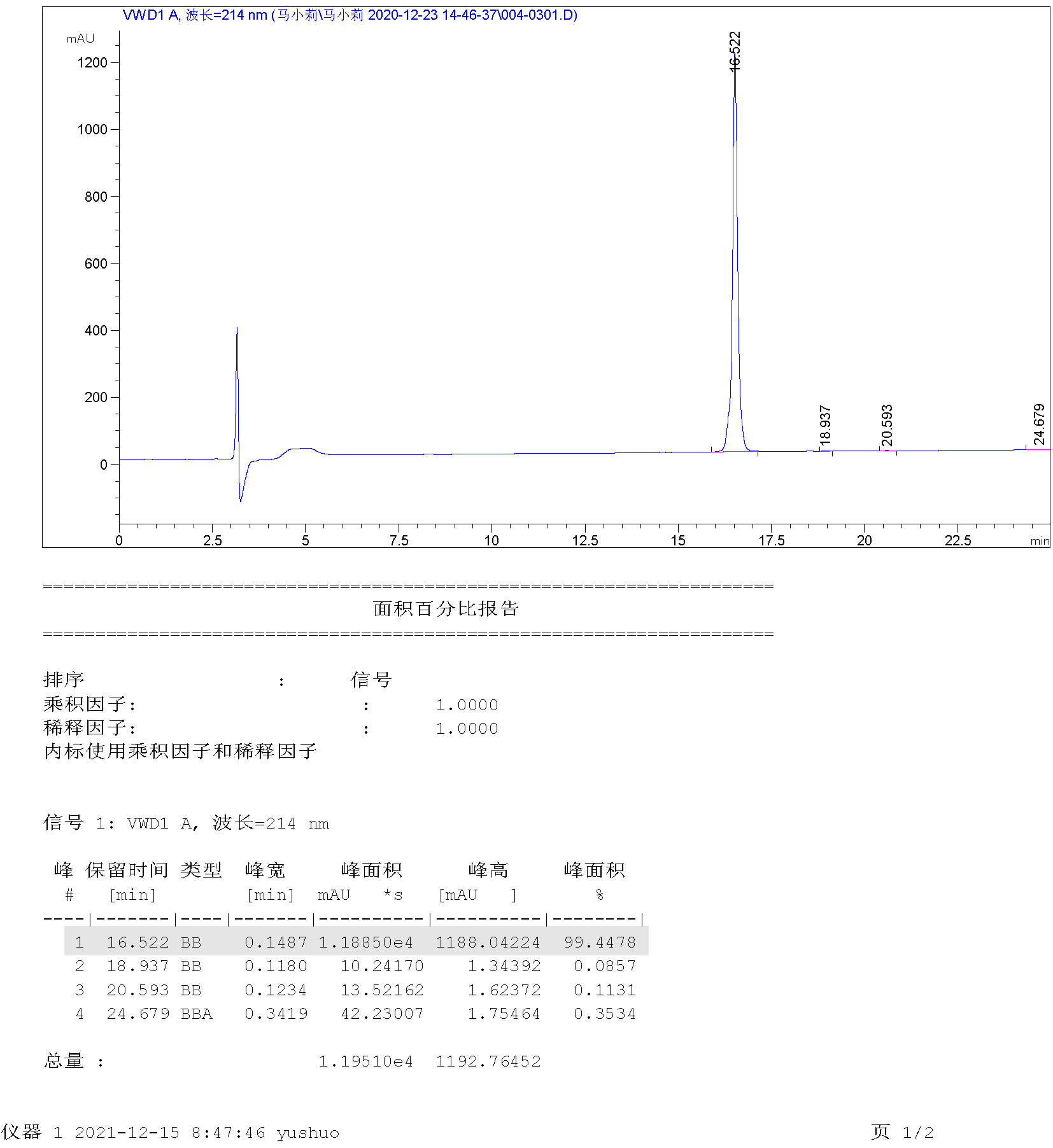


**Supplementary Figure 9.** HPLC analysis of Vt1.27[T7A]. Analytical conditions were the same as those described in Supplementary Figure 5.


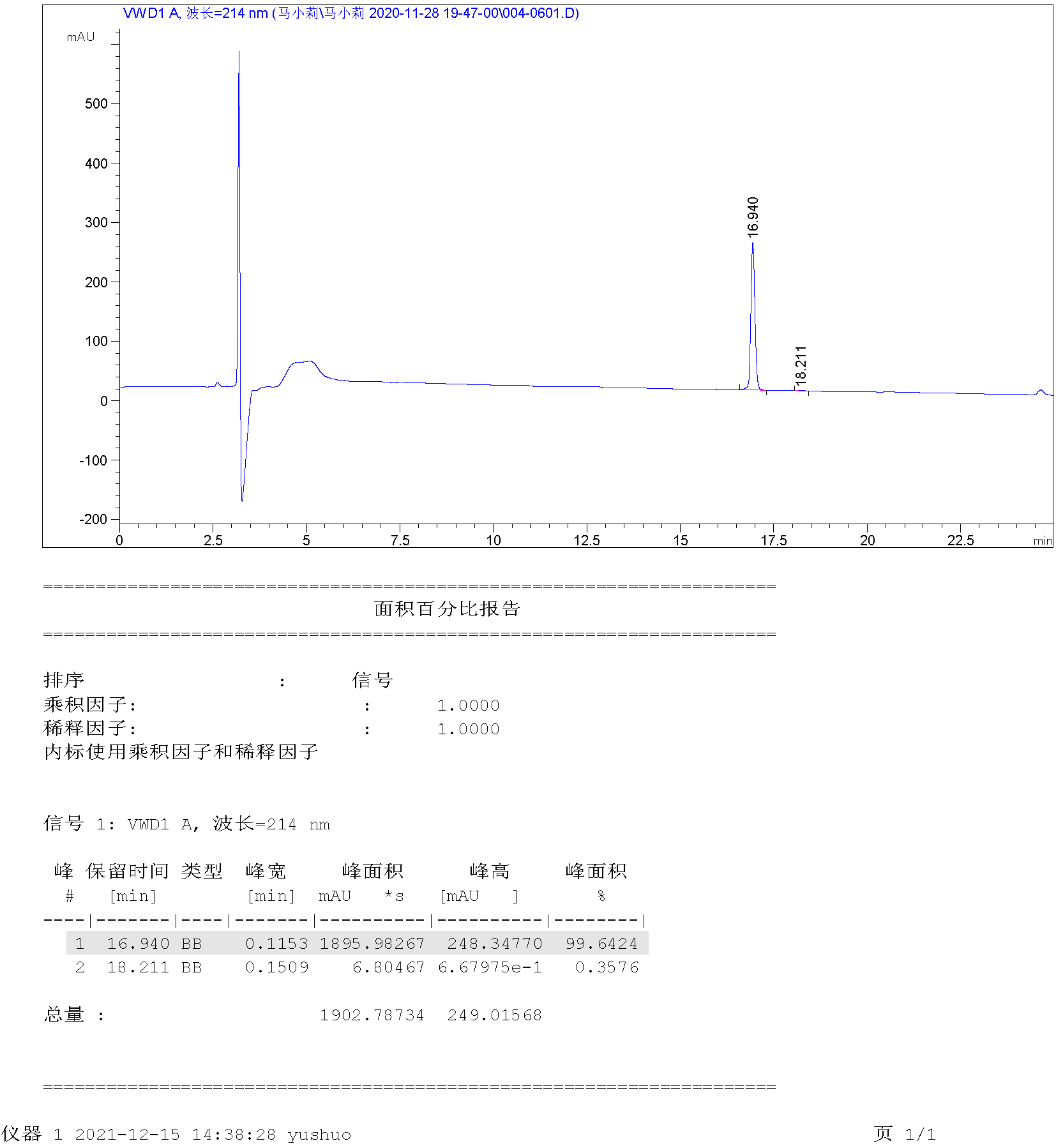


**Supplementary Figure 10.** HPLC analysis of Vt1.27[P9A]. Analytical conditions were the same as those described in Supplementary Figure 5.


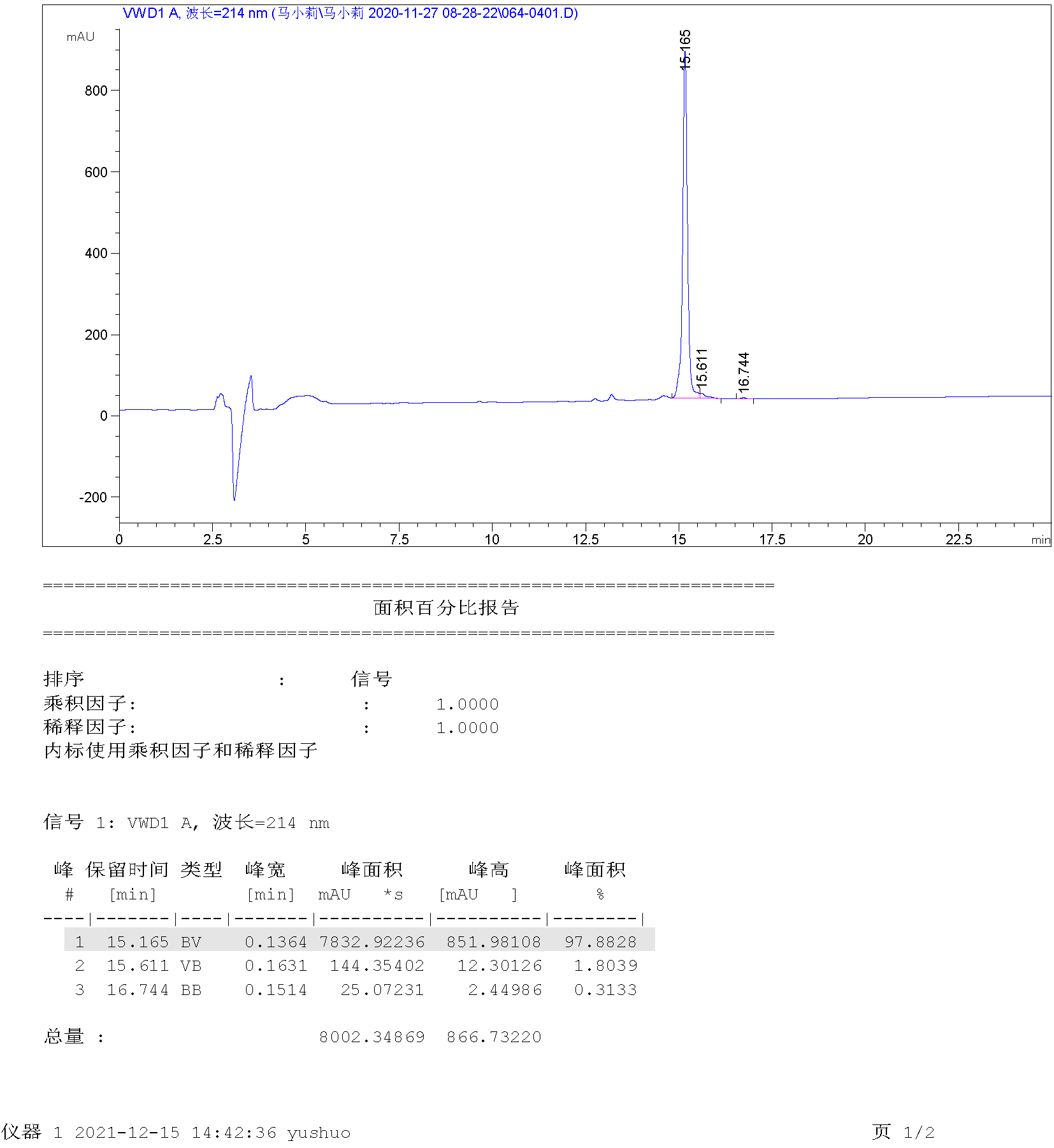


**Supplementary Figure 11.** HPLC analysis of Vt1.27[I10A]. Analytical conditions were the same as those described in Supplementary Figure 5.


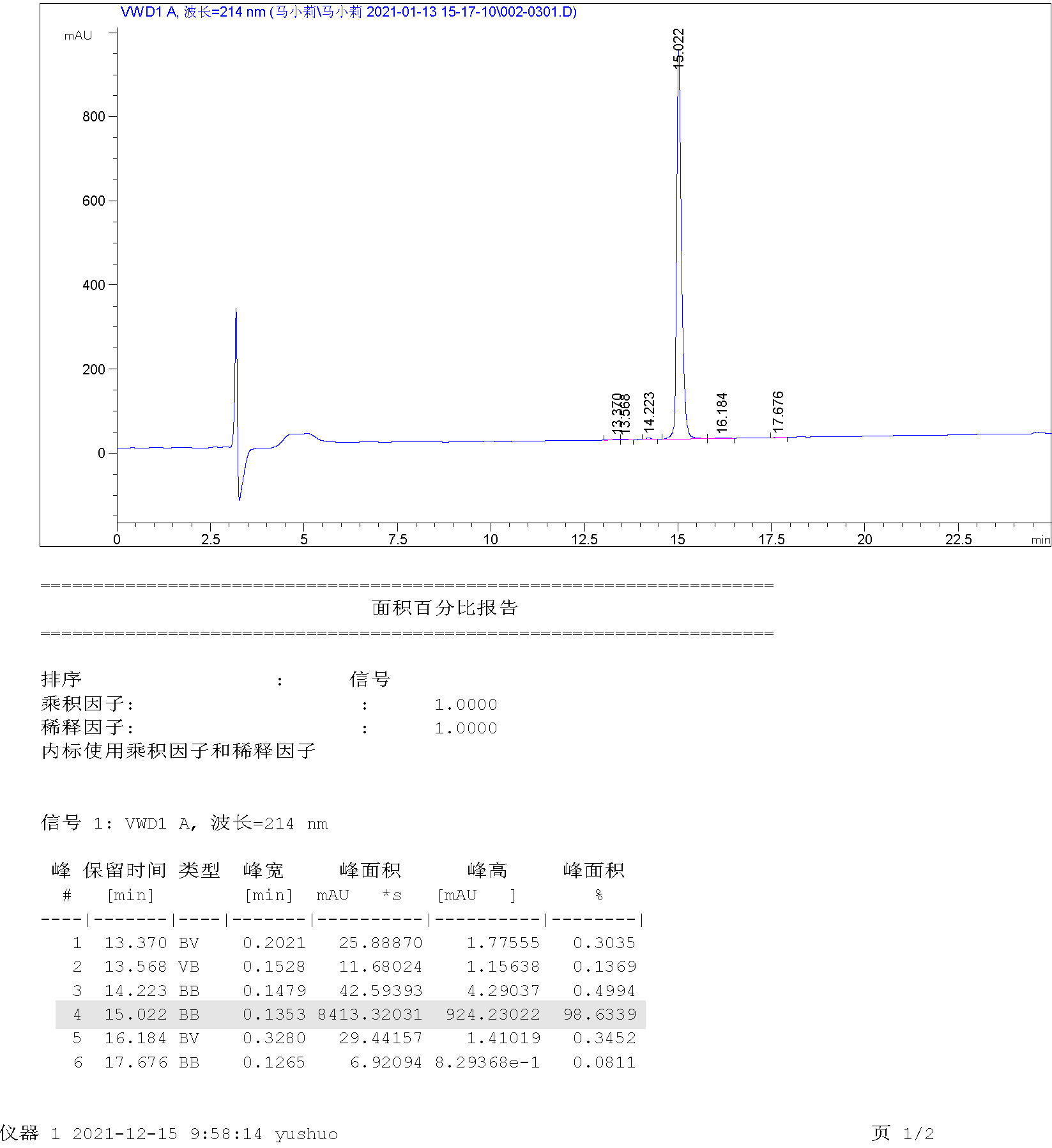


**Supplementary Figure 12.** HPLC analysis of Vt1.27[D11A]. Analytical conditions were the same as those described in Supplementary Figure 5.


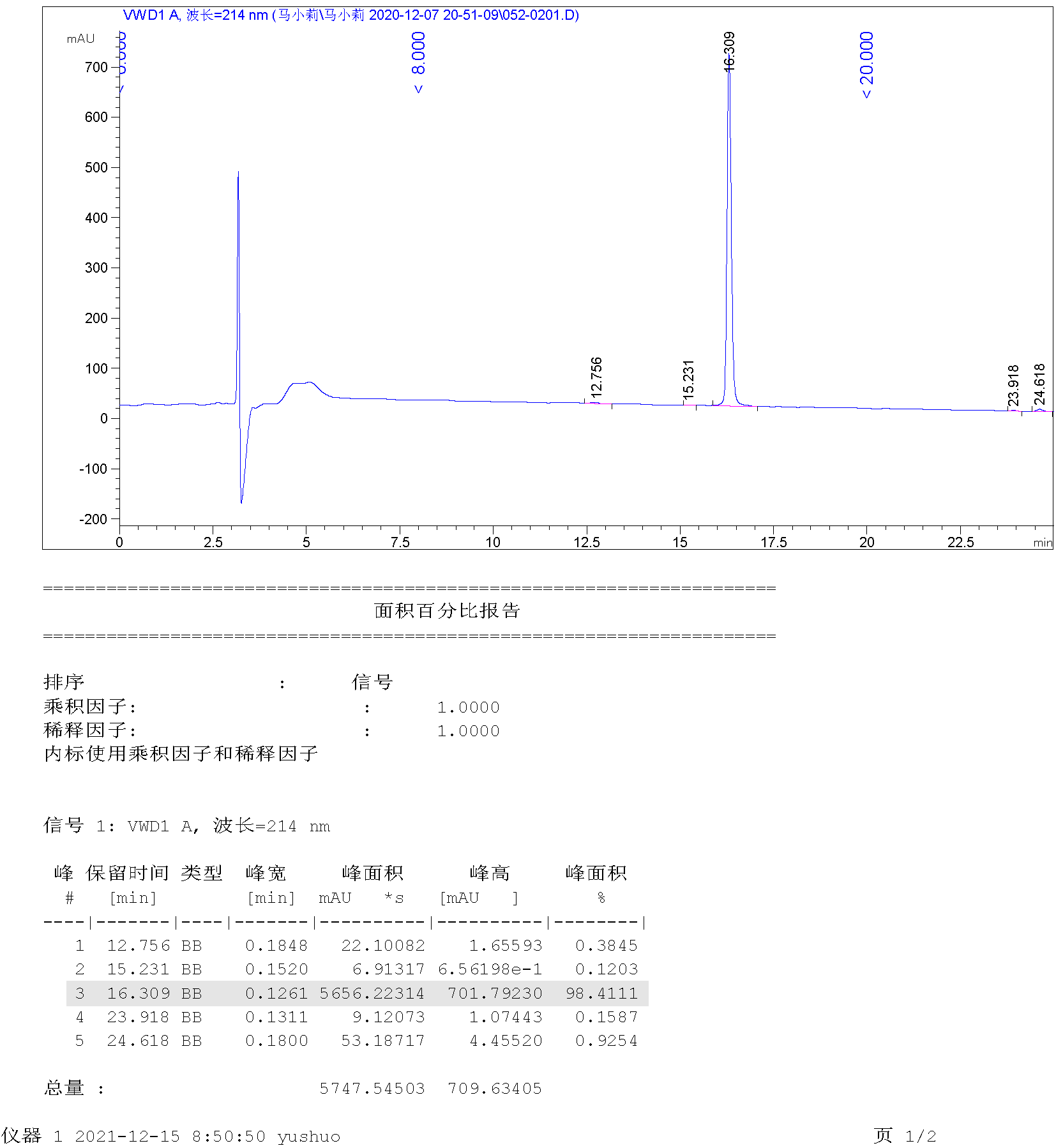


**Supplementary Figure 13.** HPLC analysis of Vt1.27[Y12A]. Analytical conditions were the same as those described in Supplementary Figure 5.


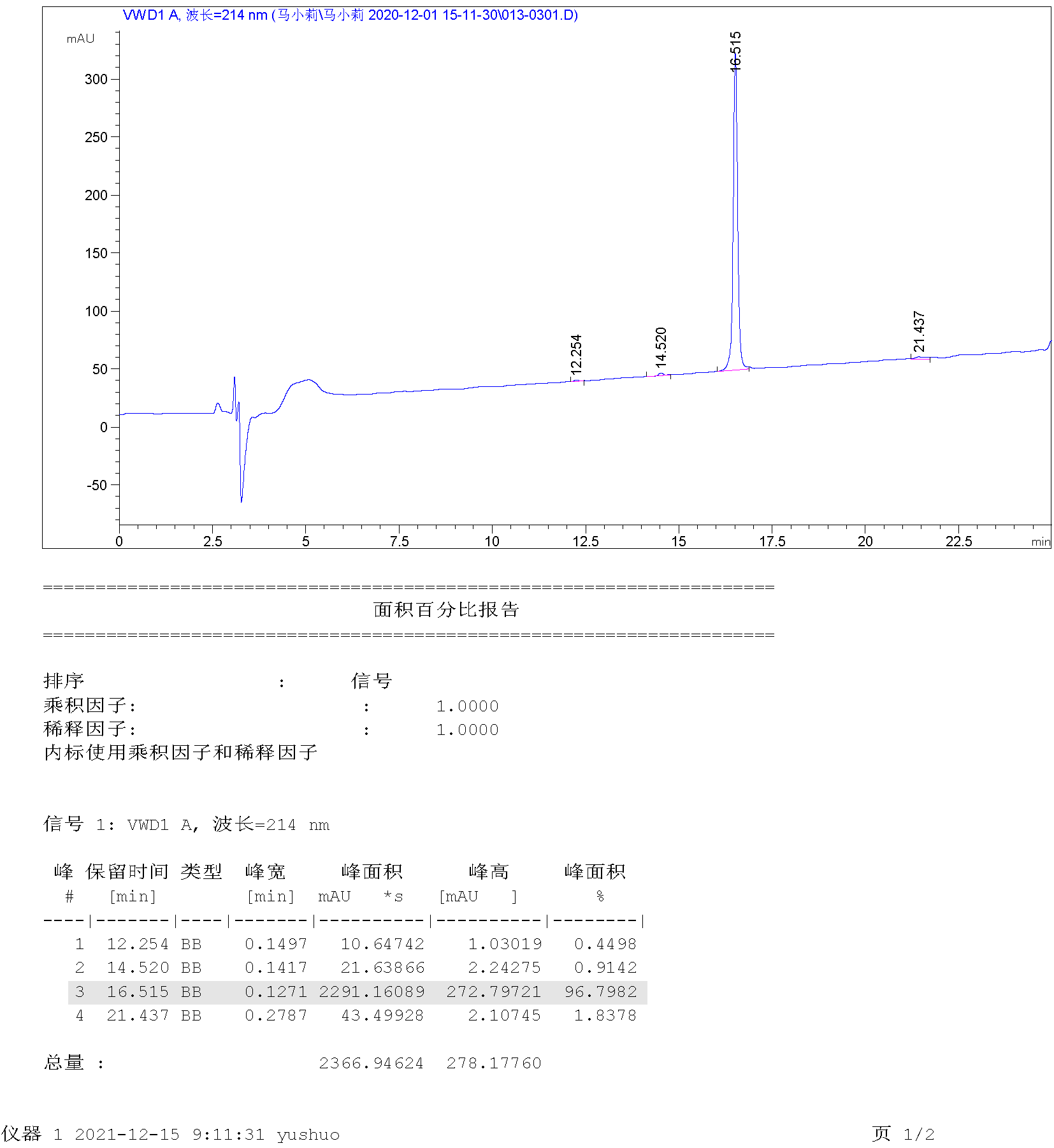


**Supplementary Figure 14.** HPLC analysis of Vt1.27[S13A]. Analytical conditions were the same as those described in Supplementary Figure 5.


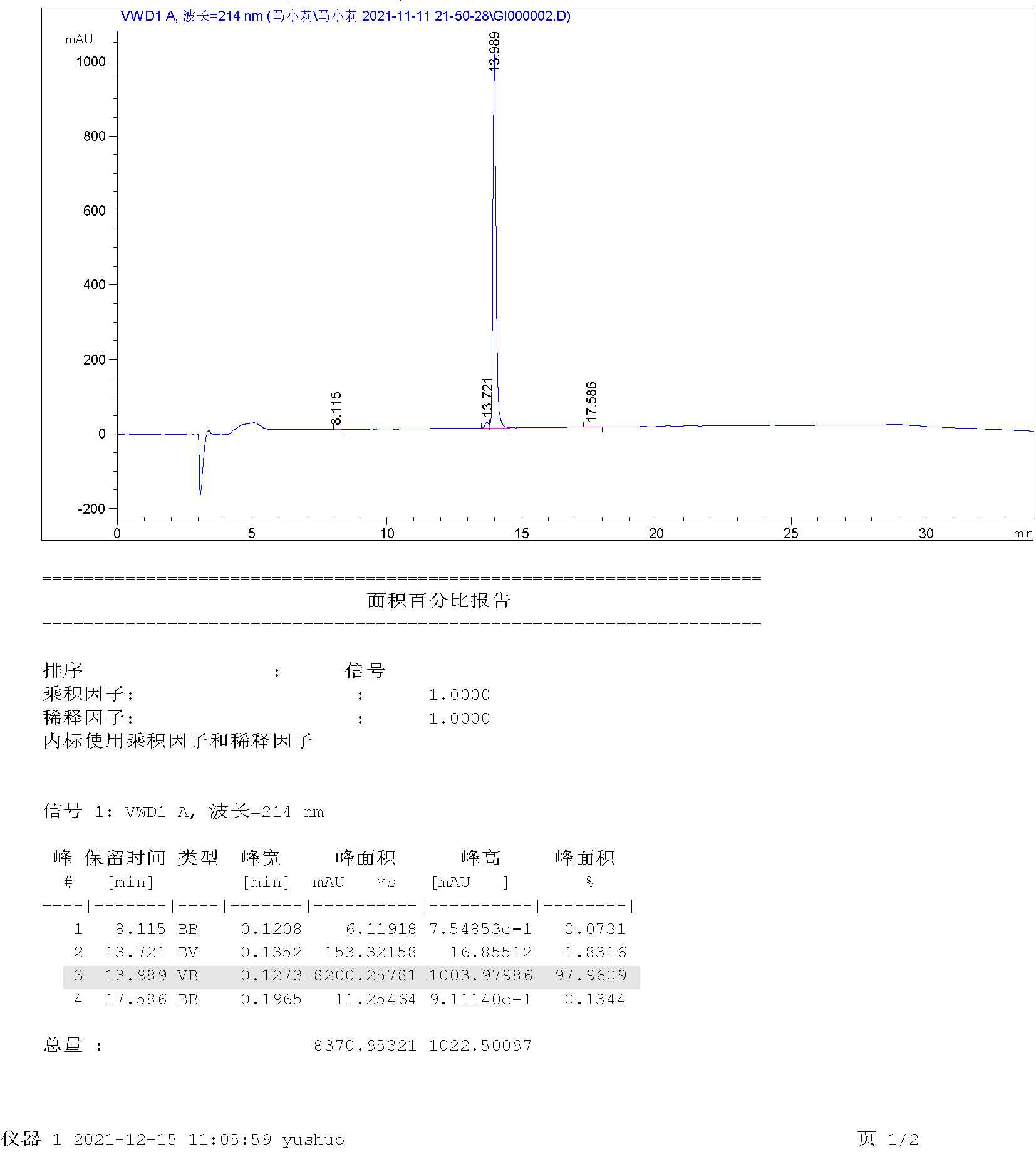


**Supplementary Figure 15**. HPLC analysis of Vt1.27[S13K]. Analytical conditions were the same as those described in Supplementary Figure 5.


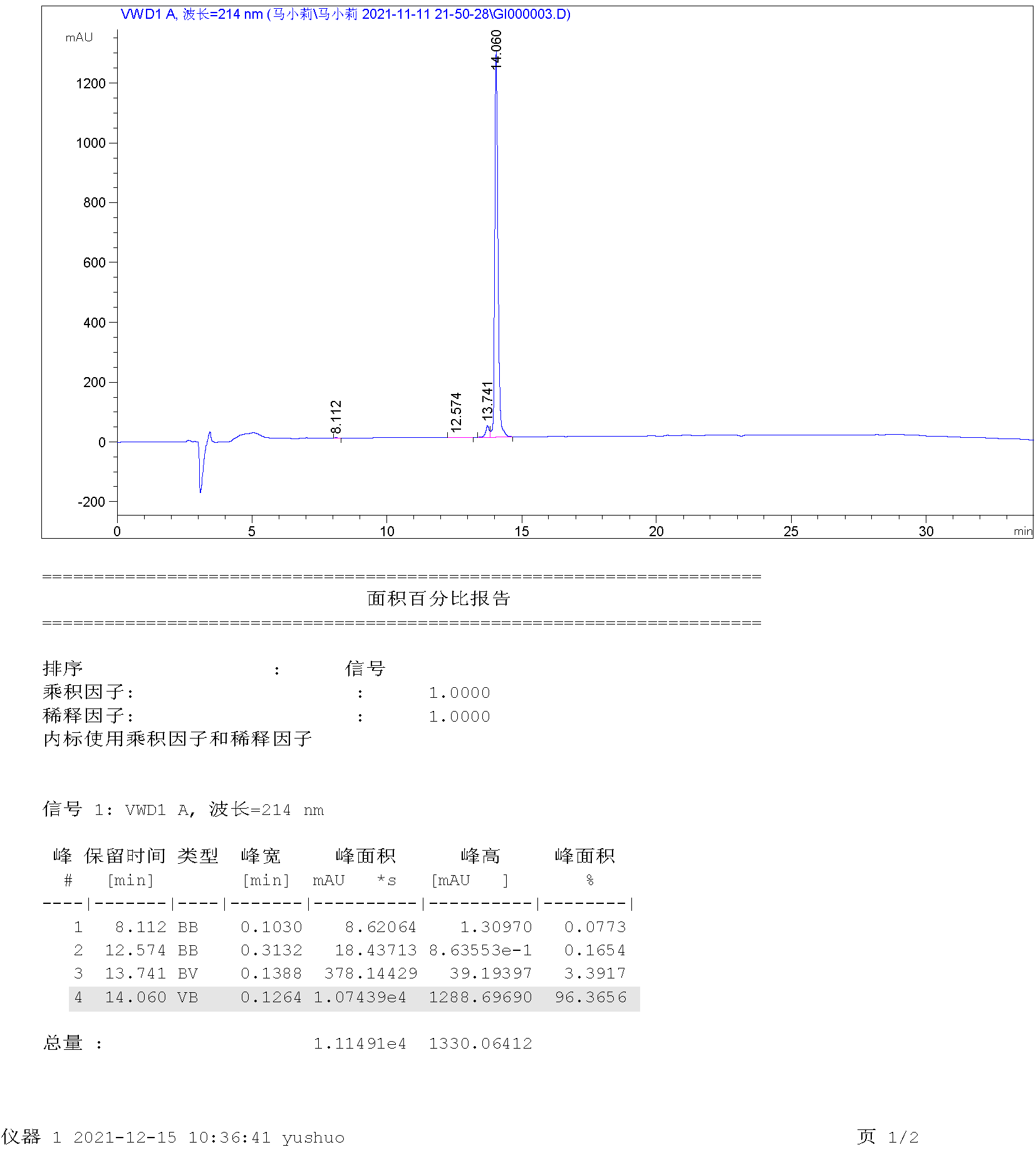


**Supplementary Figure 16.** HPLC analysis of Vt1.27[H6K,S13K]. Analytical conditions were the same as those described in Supplementary Figure 5.


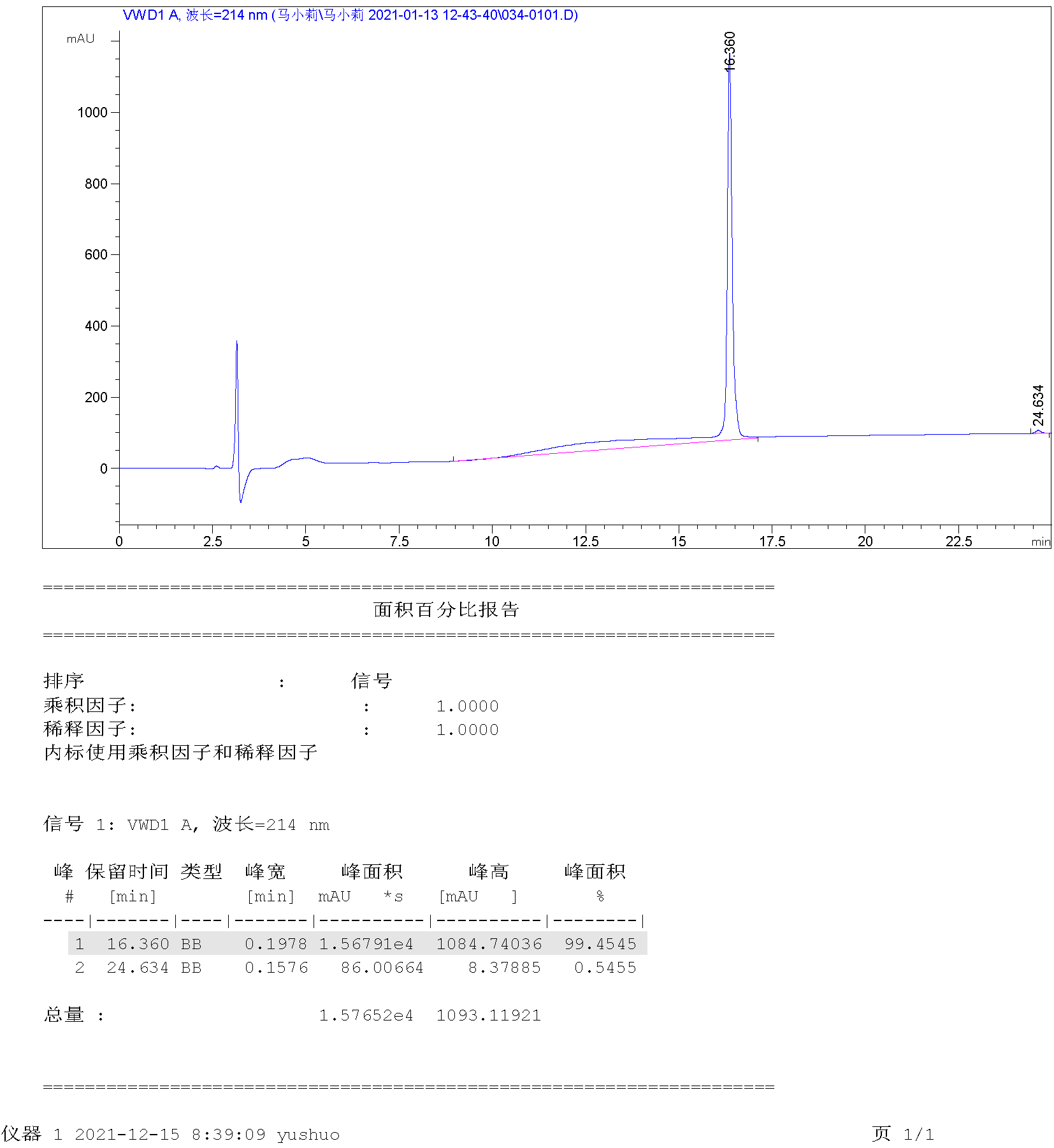


**Supplementary Figure 17.** HPLC analysis of Vt1.27[R14A]. Analytical conditions were the same as those described in Supplementary Figure 5.


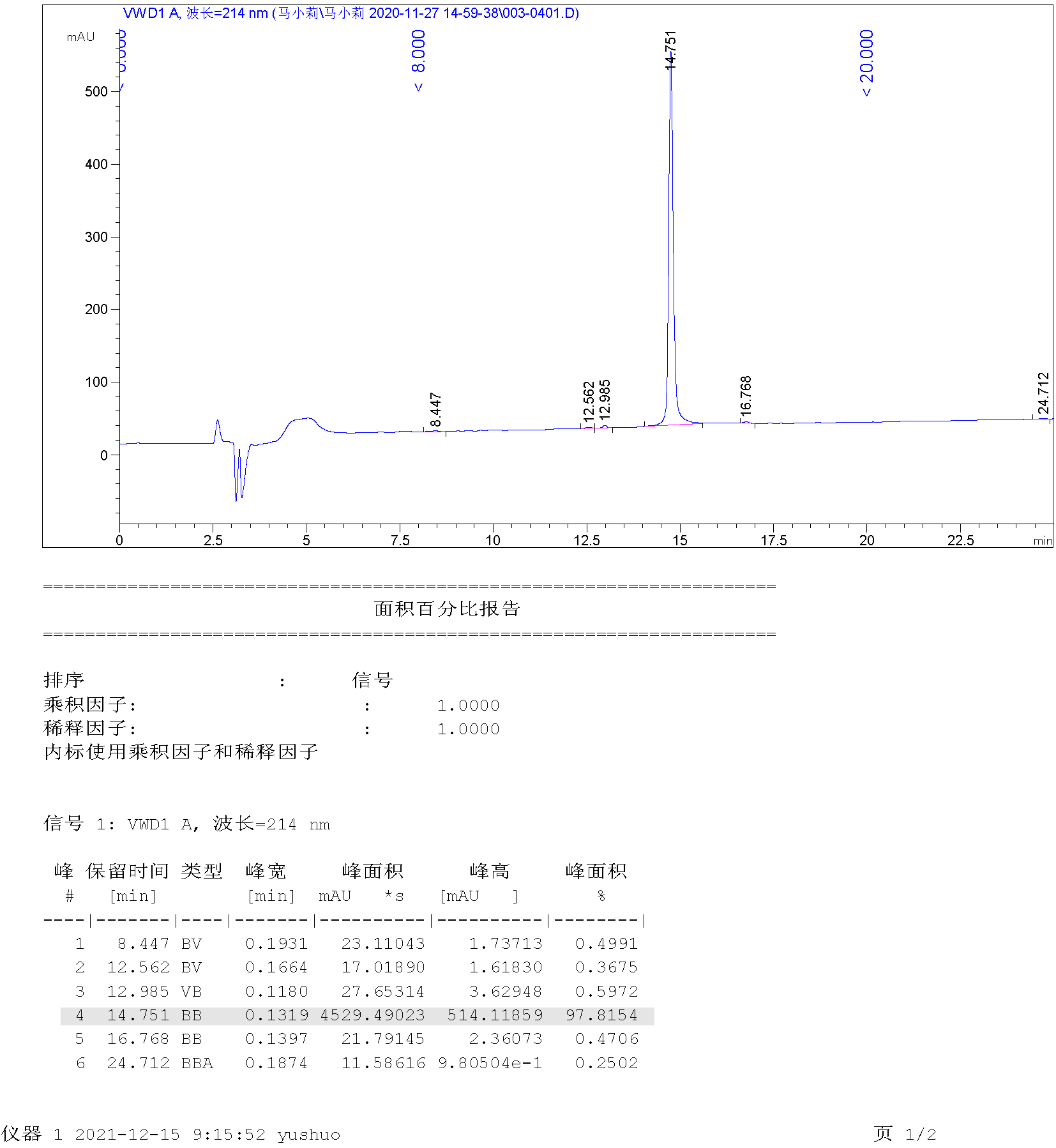


**Supplementary Figure 18.** HPLC analysis of Vt1.27[F15A]. Analytical conditions were the same as those described in Supplementary Figure 5.

**Supplementary Figure 19.** Mass spectrometry of Vt1.27(ESI) (Theoretical MW 2047.79)

**Supplementary Figure 20.**  Mass spectrometry of Vt1.27[N1A](MALDI-TOF) (Theoretical MW 2004.79)

**Supplementary Figure 21.** Mass spectrometry of Vt1.27[M4A](MALDI-TOF) (Theoretical MW 1987.79)

**Supplementary Figure 22.**  Mass spectrometry of Vt1.27[F5A](MALDI-TOF) (Theoretical MW 1971.76)

**Supplementary Figure 23.** Mass spectrometry of Vt1.27[H6A](ESI) (Theoretical MW 1981.77)

**Supplementary Figure 24.** Mass spectrometry of Vt1.27[T7A](MALDI-TOF) (Theoretical MW 2017.78)

**Supplementary Figure 25.** Mass spectrometry of Vt1.27[P9A](MALDI-TOF) (Theoretical MW 2021.78)

**Supplementary Figure 26.** Mass spectrometry of Vt1.27[I10A](MALDI-TOF) (Theoretical MW 2005.75)

**Supplementary Figure 27.** Mass spectrometry of Vt1.27[D11A](ESI) (Theoretical MW 2003.80)

**Supplementary Figure 28.**  Mass spectrometry of Vt1.27[Y12A](MALDI-TOF) (Theoretical MW 1955.77)

**Supplementary Figure 29.**  Mass spectrometry of Vt1.27[S13A](MALDI-TOF) (Theoretical MW 2031.80)

**Supplementary Figure 30.**  Mass spectrometry of Vt1.27[S13K](ESI) (Theoretical MW 2088.86)

**Supplementary Figure 31.**  Mass spectrometry of Vt1.27[H6K,S13K](ESI) (Theoretical MW 2079.89)

**Supplementary Figure 32.** Mass spectrometry of Vt1.27[R14A](ESI) (Theoretical MW 1962.73)

**Supplementary Figure 33.** Mass spectrometry of Vt1.27[F15A](MALDI-TOF) (Theoretical MW 1971.76)
